# Construction and optimization of gene expression signatures for prediction of survival in two-arm clinical trials

**DOI:** 10.1186/s12859-020-03655-7

**Published:** 2020-07-25

**Authors:** Joachim Theilhaber, Marielle Chiron, Jennifer Dreymann, Donald Bergstrom, Jack Pollard

**Affiliations:** 1 Sanofi Oncology, 270 Albany Street, Cambridge, MA 02139 USA; 2 Sanofi Oncology, Centre de Recherche de Vitry-Alfortville, 13 Quai Jules Guesde, 94400 Vitry-sur-Seine, France; 3 Relay Therapeutics, 399 Binney St, Cambridge, MA 02139 USA

**Keywords:** Predictive signature, Predictive biomarker, Gene expression profiling, Multivariate cox models, Metastatic CRC, Metastatic TNBC, Two-arm clinical trials, Aflibercept

## Abstract

**Background:**

Gene expression signatures for the prediction of differential survival of patients undergoing anti-cancer therapies are of great interest because they can be used to prospectively stratify patients entering new clinical trials, or to determine optimal treatment for patients in more routine clinical settings. Unlike prognostic signatures however, predictive signatures require training set data from clinical studies with at least two treatment arms. As two-arm studies with gene expression profiling have been rarer than similar one-arm studies, the methodology for constructing and optimizing predictive signatures has been less prominently explored than for prognostic signatures.

**Results:**

Focusing on two “use cases” of two-arm clinical trials, one for metastatic colorectal cancer (CRC) patients treated with the anti-angiogenic molecule aflibercept, and the other for triple negative breast cancer (TNBC) patients treated with the small molecule iniparib, we present derivation steps and quantitative and graphical tools for the construction and optimization of signatures for the prediction of progression-free survival based on cross-validated multivariate Cox models. This general methodology is organized around two more specific approaches which we have called subtype correlation (subC) and mechanism-of-action (MOA) modeling, each of which leverage a priori knowledge of molecular subtypes of tumors or drug MOA for a given indication. The tools and concepts presented here include the so-called differential log-hazard ratio, the survival scatter plot, the hazard ratio receiver operating characteristic, the area between curves and the patient selection matrix. In the CRC use case for instance, the resulting signature stratifies the patient population into “sensitive” and “relatively-resistant” groups achieving a more than two-fold difference in the aflibercept-to-control hazard ratios across signature-defined patient groups. Through cross-validation and resampling the probability of generalization of the signature to similar CRC data sets is predicted to be high.

**Conclusions:**

The tools presented here should be of general use for building and using predictive multivariate signatures in oncology and in other therapeutic areas.

## Background

In the past several years prediction of the response and survival of patients undergoing anti-cancer therapies, using machine learning models based on gene expression profiling of tumor tissues, has been of great interest. These modeling efforts have led to many context-dependent statistical models, typically relying on a subset of the genes profiled, and which are loosely referred to as signatures or biomarkers. From the outset, an important distinction has been made between purely “prognostic” signatures, which predict outcome under a single treatment regimen (such as, for instance, breast cancer and a single type of hormone therapy), and “predictive” signatures, which are able to predict differential outcomes, i.e. between treatments involving different drug regimens. The latter type of signature might ultimately be considered more important, because it provides a criterion for choosing one drug regimen over another, and hence for optimizing the treatment of patients in actual clinical settings. However, signatures derived so far have overwhelmingly been of the prognostic type, principally because much of the underlying data has arisen from one-arm clinical trials. In these studies (e.g. [1–6] for breast cancer) the therapeutic effects of the drug are confounded with the natural spectrum of patient responses, and even bringing a priori knowledge to bear, it is usually very difficult to interpret the prognostic signature as a predictor of drug response. On the other hand, gene expression profiling studies involving two-arm clinical trials have been rarer (e.g. [7, 8]), and the methodology for deriving predictive signatures less prominent.

In this context, we were recently brought to analyze gene expression and associated clinical outcome data for some two-arm clinical trials, including one [9] targeting late-stage metastatic colorectal cancer (CRC) and designed to test an anti-angiogenic molecule, aflibercept [10, 11], and another [12, 13] targeting triple negative breast cancer (TNBC), using iniparib, a small molecule inducer of oxidative stress. On the basis of these data, we have been able to generate gene expression signatures which enable stratification of the CRC or TNBC patients into groups which experience quantifiably different progression free survival (PFS) time under treatment with aflibercept or iniparib, respectively, relative to treatment without these agents. It should be emphasized that the signatures so obtained are predictive, in that they can estimate how the *same* patient might differentially (hypothetically) fare under the two different treatment arms.

In deriving the predictive signatures to evaluate for instance the effectiveness of afllibercept for the CRC patients, building on existing approaches [14–16] we adopted a general mathematical framework and a number of computational and graphical devices which should be portable across many indications and indication-specific statistical models. The more specific feature of the statistical model used for CRC is that it is based on the CRC intrinsic molecular subtypes [17, 18], which are used to first transform the input gene expression profiles into a continuous feature space of lower dimensionality, an approach we have termed subtype correlation (subC). For TNBC we have adopted another starting approach which we have termed MOA modeling, which is based on the simple expedient of restricting genes to the presumed mechanism of action of iniparib, namely genes involved in oxidative stress response. However we emphasize that the general mathematical framework presented here is independent of the details of subC or MOA, and starts with the concept of the differential log hazard ratio (dLHR) as the main biomarker of interest [14]. The computational and graphical devices include survival scatter plots, for graphically emphasizing the predictive power of the biomarker; the hazard ratio receiver operating characteristic (hROC), which shows the tradeoff between the stringency of patient selection and treatment benefit to the patients; the area between curves (Abc), which enables model optimization; and the patient selection matrix (PSM), which numerically summarizes the consequences of specific assignments of patients to predicted response groups. In all, we believe that these “use cases” provides good examples of systematic signature construction, and that the methods presented here should be of general utility to those engaged in predictive signature discovery.

In what follows we first focus on signature derivation for the CRC patients, before covering in a more abbreviated way a similar but not identical analysis carried out for the TNBC clinical trial.

## Results

### Experimental design for the AFLAME two-arm clinical trial

The CRC data analyzed was generated by a phase 3 two-arm clinical trial called AFLAME [9], conducted to test the efficacy of the anti-angiogenic, biologic drug aflibercept [10], in combination with standard-of-care chemotherapy (FOLFIRI panel [19]), for patients with metastatic colorectal cancer. In the trial, patients were randomly assigned in 1:2 ratio to the two treatment arms, the first using FOLFIRI alone (the “placebo” arm), and the second with FOLFIRI augmented by aflibercept (the “aflibercept” arm). Out of the total of *n* = 332 patients with clinical outcome data (109:223 placebo:aflibercept assignment ratio), for *n* = 238 patients, archival, formalin-fixed paraffin-embedded (FFPE) samples of colorectal tissue derived from the original patient biopsies were profiled for gene expression quantification through RNA-sequencing (RNA-seq) on the Illumina HiSeq 2000 platform [20]. For the analyses described here a subset of the data consisting of *n* = 209 gene expression profiles (68:141 placebo:aflibercept ratio), obtained after quality-control of samples for tumor content and quality of RNA-seq profiling (see below) was used.

Associated clinical information was available for almost all of the trial subjects. Measured clinical variables for each subject included assigned treatment arm, progression-free survival (PFS) time and censoring status, corresponding values for overall survival (OS) time, and objective response (OR). Overall, significant increase in PFS for aflibercept relative to placebo was observed, with computed hazard ratio hR = 0.618 [0.48, 0.79]_0.95_ and *P*-value pR = 2.8 × 10^− 4^ (log-ranks test) obtained from analysis for all *n* = 332 patients. The *n* = 209 subset of these patients with high-quality gene expression profiles exhibited a smaller aflibercept to placebo hazard ratio hR = 0.486 [0.35, 0.67]_0.95_ (pR = 2.8 × 10^− 4^) which however was not statistically significantly different from that obtained for the entire cohort of *n* = 332 patients (hR = 0.486 falls within the 95% confidence interval of the distribution inferred for the larger population; *P*-value = 0.062), and thus reflected normal variance in sampling from the parent population.

The focus of the regression models presented here was in prediction of PFS.

### Data set assembly and pre-processing

Raw RNA-seq data (FASTQ files) for each of the *n* = 238 patient samples with matched outcome data in the AFLAME corpus were processed by computer by sequentially applying the Star aligner [21] and Cufflinks transcript-abundance estimation [22] algorithms, generating signal estimation for 26,775 genes for each sample. Quality control was then performed, by retaining only profiles with at least minimum tumor content in the original sample, by eliminating profiles with low RNA-seq read statistics, and by removing outliers as detected in a subsequent principal components analysis. The remaining *n* = 209 gene expression profiles were then quantile-normalized together [23] to create a single data matrix. Expression values were individually log2-transformed, and batch effects removed by using the batch correction algorithm ComBat [24]. Finally, gene expression data was standardized by mean subtraction and division by standard deviation for each gene independently: more specifically, if **X** refers to the {p × n} gene expression data matrix after log_2_ transformation, with rows *i = 1, …, p* corresponding to genes, and columns *j = 1, …, n* to samples (*p* = 26,775, *n* = 209), then the expression values *x*_*ij*_ for gene *i* were standardized into values *y*_*ij*_ according to the equation

1 $$ {y}_{ij}=Z\left({x}_{ij}\right)\equiv \frac{x_{ij}-{\overline{x}}_i}{s_i},\kern0.36em j=1,\dots, n, $$

where ${\overline{x}}_i$ and *s*_*i*_ are the mean value and sample standard deviation of *x*_*ij*_ across the *n* samples. The final result was an {n × p} = {209 × 26,775} data matrix **Y**, of normalized and standardized gene expression values, which was the starting point of the analyses presented below (see Additional files 1 and 2 for the non-standardized gene expression data matrix and the corresponding clinical metadata, respectively).

### Multivariate cox regression models for two-arm clinical studies

To build a predictive signature, we used multivariate Cox proportional hazard models [25, 26] to express the statistical dependence of patient survival time on both gene expression and treatment arm. For a patient with gene expression vector **x** (which for the CRC example has been standardized in accordance to Eq.(1)) the models were of the form

2 $$ \log \left(\frac{\lambda \left(t|z,\mathbf{x}\right)}{\lambda_0(t)}\right)={\beta}_0z+\sum \limits_{l=1}^K{\beta}_l\cdot {\tilde{x}}_l+z\sum \limits_{l=1}^K{\gamma}_l\cdot {\tilde{x}}_l, $$

where *λ*(*t*| *z*, **x**) is the hazard function (or risk per unit time) at time *t*, for the individual with covariate vector (*z*, **x**), *λ*_0_(*t*) the baseline hazard function (the hazard which applies to an individual with all covariates exactly equal to 0), and where *z* is a binary indicator of treatment arm, with *z* = 0 for the control treatment arm and *z* = 1 for the aflibercept treatment arm. The symbol **x** indicates the entire gene expression vector (here of dimension *p* = 26,775), while the variables $\tilde{x}_{l},\kern0.36em l=1,\dots, K,$ for some *K* « *p*, refer to reduced-dimensionality covariates which are obtained from **x** using the CRC intrinsic subtypes, as explained shortly below. *t* refers to PFS time, here expressed in units of months.

The left-hand side of Eq.(2) is equal to the log-hazard-ratio, which for brevity we denote by the symbol

3 $$ \xi \left(z,\mathbf{x}\right)=\log \left(\frac{\lambda \left(t|z,\mathbf{x}\right)}{\lambda_0(t)}\right). $$

On the right-hand side of Eq.(2) the set of variables *β*_*0*_, and {*β*_*l*_, *γ*_*l*_}, *l* = *1, …, K* are the Cox model coefficients, with the symbols *β* and *γ* representing direct and interaction effects, respectively. As will be explained below, the interaction terms are central in the prediction of optimal treatment for a given patient.

### Projection onto the CRC subtype centroids generates a dimensional reduction

Because of the high dimensionality of the gene expression data, it was essential that the models be appropriately regularized [27] through feature selection and/or transformation of selected features, effectively operating a dimensional reduction on the input feature space. This was done using a priori knowledge about colorectal cancer, in the form of existing classifications of CRC profiles into so-called “intrinsic” subtypes. Several CRC subtype classification schemes exist [18], each based on unsupervised (clustering) analyses of independent bodies of gene expression data. Most of the classification schemes are embodied by a set of reference profiles (“centroids”), each centroid within a given set defining an idealized instance of a different subtype. In a given classification scheme the centroids are defined on a generally small subset (10s to 100 s of genes) of the total collection of genes defined for a given gene expression corpus (~ 20,000 genes). While the different extant subtyping schemes are not strictly consistent in terms of the subtype memberships predicted [18], one can regard each collection of centroids as providing a small (mathematical) basis of vectors spanning the space in which gene regulation biologically important for CRC is occurring, and hence as directly providing the reduced-dimensionality feature space over which the regression models should plausibly be built.

In the present work we have used two CRC subtype classification schemes as bases for constructing the predictive signatures. These schemes are namely 1) the classification defined by Laurent-Puig and collaborators and described in Marisa et al. [17] (here labeled LP) and 2) the “consensus molecular subtypes” classification defined by Guinney et al. [18] (here labeled CMS), deriving from a consensus between six independent subtyping schemes, including the LP scheme. The corresponding signatures will be referred to as subC-LP and subC-CMS.

As a definite example of the methodology, consider the subC signature based on the LP classification (subC-LP signature). Under the LP scheme [17], a given CRC sample is classified into one of six distinct subtypes labeled {C1, …, C6}, in accordance to the centroid with which its gene expression profile has the largest correlation, the centroids being defined on a restricted set of 57 genes. The six centroids {c_1_,**.** .**.**, c_6_} defining the six LP subtypes {C1, …, C6} are given in Additional file 3 (and see Additional file 4 for the corresponding centroids for the four CMS subtypes).

Note that in generating the signature, discrete classification into subtypes is not necessary or desirable; rather the centroids directly define a set of continuous variables. Thus for a given input profile, the normalized and standardized gene expression vector **x**, with values for 26,775 genes, is transformed into a vector of 6 variables, by computing the correlation of **x** to each of the six LP centroids {**c**_1_,. .., **c**_6_}. Mathematically, **x** is transformed into a 6-dimensional vector **ζ** by the formula

4 $$ {\zeta}_j={\tanh}^{-1}\left(r\left({\mathbf{c}}_j,\mathbf{x}\right)\right),\kern0.6em j=1,\dots \kern0.36em ,6\kern0.36em , $$

where *r (****c***_*j*_*,****x****)* denotes the Pearson correlation coefficient of the vector **x** with the centroid **c**_j_ (where only genes overlapping between the centroid and gene expression profile components, namely in the present case 54 genes, are used). In Eq.(4) the function tanh^− 1^ implements the Fisher-transform of the correlation coefficient (a standard symmetrizing transformation [28]). In geometrical terms, Eq.(4) can be considered a (non-linear) projection of **x** onto a vector space of much lower dimensionality.

**Fig. 1:**
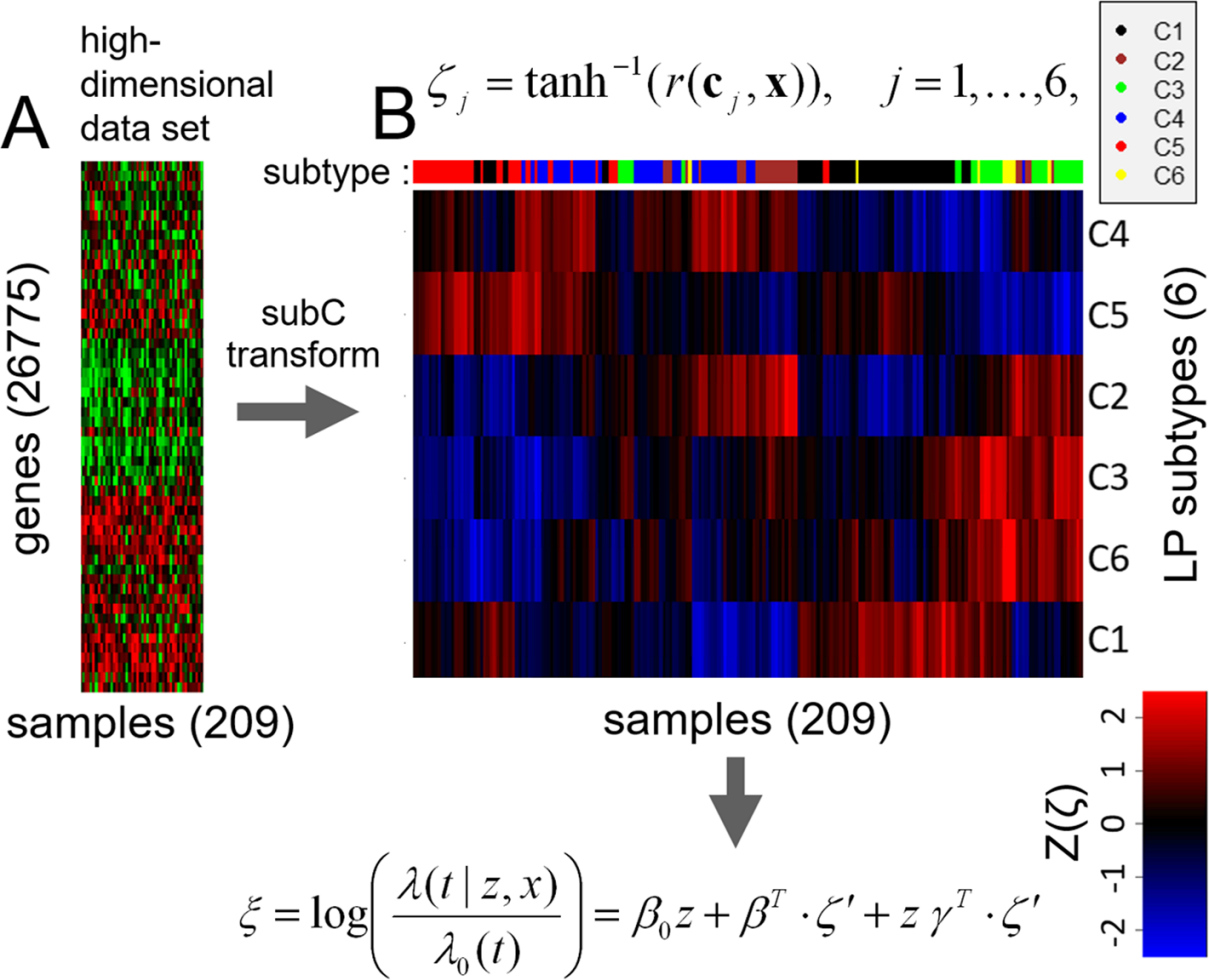
Projection onto CRC subtypes reduces the input gene expression data set to a 6-dimensional space. The example shown here is for the LP subtyping scheme defined by Marisa et al. [17]. Starting from the full gene expression matrix (**a**), transformation by Eq.(4) (reproduced at the top of figure **b**) generates the 6 × 209 matrix of ζ coefficients shown as a red-blue heat map (**b**), with the corresponding discrete subtype assignments indicated by the colored bar at the top. The subC regression model uses the ζ coefficients as covariates (Eq.(5), reproduced at the bottom of figure **b**)

A heat map of the **ζ** coefficients deriving from the *n* = 209 AFLAME data matrix is displayed in Fig. 1, showing the continuous set of low-dimensionality features on which the subC-LP regression model is built. Setting $\tilde{x}=\zeta \hbox{'}$, where the prime indicates mean-centering, the general Cox regression model of Eq.(2) becomes

5 $$ \xi \left(z,\mathbf{x}\right)=\log \left(\frac{\lambda \left(t|z,\mathbf{x}\right)}{\lambda_0(t)}\right)={\beta}_0\kern0.17em z+{\beta}^T\cdot \zeta \hbox{'}+z\kern0.17em {\gamma}^T\cdot \zeta \hbox{'}, $$

where as before *z* ∈ {0, 1} is a binary covariate indicating the treatment arm (*z* = 0 for placebo, *z* = 1 for aflibercept), *β*_*0*_ the corresponding treatment arm coefficient, and ***β*** and ***γ*** 6-dimensional vectors of coefficients for direct and interaction effects. The coefficients in Eq.(5) were estimated using standard iterative methods based on partial likelihood maximization [26] (R programming environment [29]). The resulting values for the subC-LP Cox coefficients are given in Table 1.

**Table 1:** Cox coefficients for the subC-LP or subC-CMS models trained on the AFLAME data. Values of the coefficients are indicated along with 95% confidence intervals and *P*-values

**subC-LP**				
**Component**	**beta [CI 95%]**	**Pbeta**	**gamma [CI 95%]**	**Pgamma**
beta0	−0.79 [−1.12, − 0.45]	4.20E-06		
C1	−1.77 [−6.79, 3.24]	4.90E-01	6.48 [0.73, 12.22]	2.70E-02
C2	−1.21 [−3.83, 1.41]	3.70E-01	2.56 [−0.43, 5.54]	9.30E-02
C3	3.49 [−0.44, 7.42]	8.20E-02	−5.94 [−10.35, − 1.53]	8.30E-03
C4	−0.19 [−4.75, 4.36]	9.30E-01	2.79 [−2.55, 8.13]	3.10E-01
C5	3.11 [−2.57, 8.78]	2.80E-01	−8.89 [−15.63, − 2.15]	9.70E-03
C6	0.75 [−2.52, 4.02]	6.50E-01	−3.41 [−7.25, 0.43]	8.20E-02
**subC-CMS**				
**Component**	**beta [CI 95%]**	**Pbeta**	**gamma [CI 95%]**	**Pgamma**
beta0	−0.75 [−1.08, − 0.42]	7.90E-06		
CMS1	5.81 [−12.96, 1.35]	1.10E-01	17.18 [6.91, 27.46]	1.00E-03
CMS2	6.29 [−14.94, 2.36]	1.50E-01	19.15 [6.52, 31.78]	3.00E-03
CMS3	−3.07 [−9.42, 3.27]	3.40E-01	12.02 [2.86, 21.18]	1.00E-02
**CMS4**	**5.38 [−13.52, 2.76]**	2.00E-01	**16.79 [4.75, 28.83]**	**6.30E-03**

### Differential log-hazard-ratio as a predictive biomarker

Based on the fitted model of Eq.(5), for a given patient, we define the differential log-hazard-ratio (dLHR) ∆ξ as the logarithm of the hazard-ratio of the aflibercept arm to that of the control arm. For gene expression vector **x**, ∆ξ is given by the expression

6 $$ \Delta \xi \left(\mathbf{x}\right)=\log \left(\frac{\lambda \left(t|z=1,\mathbf{x}\right)}{\lambda \left(t|z=0,\mathbf{x}\right)}\right)=\xi \left(z=1,\mathbf{x}\right)-\xi \left(z=0,\mathbf{x}\right), $$

where ξ(z, **x**) is given in Eq.(3). By definition of the hazard functions, patients with ∆ξ(**x**) < 0 should have generally better survival in the aflibercept arm than in the control arm, and conversely for patients with ∆ξ(**x**) > 0. Thus if the model underlying the calculation of ∆ξ is validated, ∆ξ(**x**) can then be used as a “biomarker” for selecting optimal treatment for a given patient [14].

**Fig. 2:**
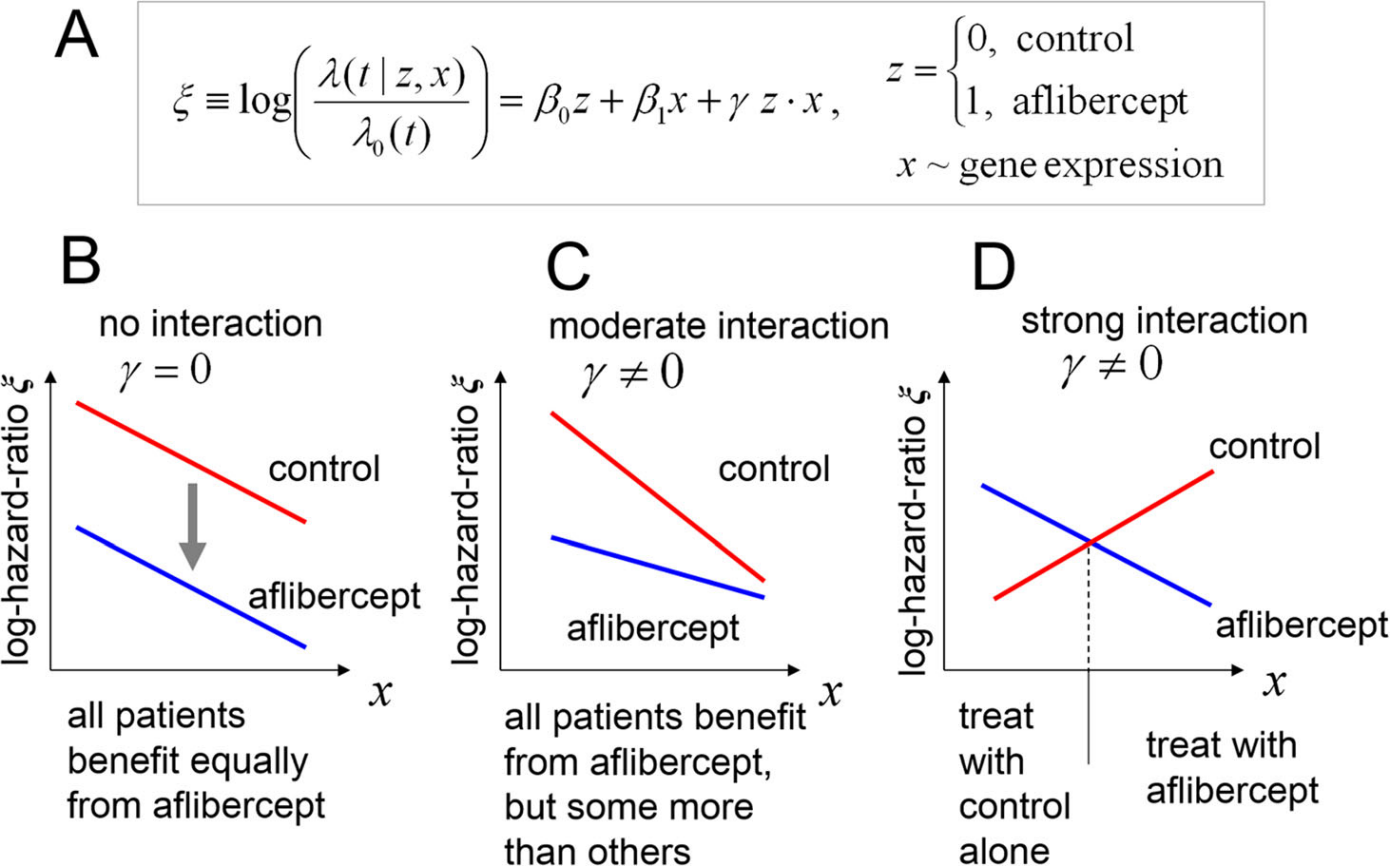
Role of the {treatment-arm × gene expression} interaction term in the Cox models. **a** Schematic equation for the log-hazard-ratio ξ as a function of treatment arm and gene expression, showing both direct and interaction terms. **b** ‘no interaction’ case: log-hazard-ratio profiles in the absence of interaction effects (γ = 0), with β_0_, β_1_ < 0: the aflibercept treatment arm is always favored, and the signature is only prognostic, with the same dependence on gene expression in both treatment arms. **c** ‘moderate interaction case’: with γ > 0 and β_0_, β_1_ < 0, and the range of *x* indicated, all patients benefit from aflibercept but some more than others. **d** ‘strong interaction case’: with γ < 0, β_0_ < 0 and | γ | > β_1_ > 0, the optimal treatment may ‘flip’ depending on *x*

Using Eq.(5) in Eq.(6) we have

7 $$ \Delta \xi \left(\mathbf{x}\right)={\beta}_0+{\gamma}^T\cdot \zeta \hbox{'}, $$

so that the dependence of ∆ξ(**x**) on gene expression arises entirely from the multivariate interaction terms *γ*_*l*_, l = 1,. .., 6. Although the structure of Eq.(6) is simple, qualitatively different predictive outcomes are possible depending on the signs and values of the interaction terms. This is illustrated in Fig. 2, where we show in schematic form a multivariate Cox model with treatment, gene expression and interaction effects (Fig. 2a, Cox coefficients β_0_, β_1_ and γ respectively). For this model, three qualitatively distinct scenarios are possible: i) if the interaction term is 0 (Fig. 2b, γ = 0 with say β_0_, β_1_ < 0, ‘no interaction’ case), the lines depicting the log-hazard-ratios for the patients in the two treatment arms are parallel, ∆ξ(x) = β_0_ = constant < 0, and the aflibercept arm is always equally favored; ii) on the other hand, if the interaction term is non-zero (Fig. 2c, with say γ > 0, β_0_, β_1_ < 0 and |γ| < |β_1_ |, ‘moderate interaction’ case), for the range depicted the aflibercept arm is still always favored, but some patients will benefit markedly more than others, iii) finally, if the interaction term is non-zero and large (Fig. 2d, with say γ < 0, β_0_, β_1_ > 0 and |γ| > |β_1_ |, ‘strong interaction’ case), the lines depicting the log-hazard-ratios may cross, splitting the prospective patient population into two groups, each favored by a different treatment arm.

Figure 2b-d also illustrates the difference between prognostic and predictive biomarkers. In all cases gene expression is strongly prognostic of patient survival: thus the prognostic biomarker ξ indicates that patients in a *given* treatment arm may exhibit widely varying survival times. On the other hand, the predictive biomarker ∆ξ focuses on *comparison* of the two treatment arms, and in some cases may vary little (as in Fig. 2b), despite strong variation in the two treatment arms taken separately.

### Model cross-validation

To robustly estimate the predictive performance of ∆ξ, 5-fold cross-validation was applied throughout. In this procedure [15, 30, 31], the set of *n* = 209 samples was first randomly divided into five equal “folds” of approximately 42 samples each, one fold then being removed at a time to constitute an on-the-fly test set, and the remainder of the data being used as a training set, for which the model variables were computed. The differential log-hazard-ratios ∆ξ were then computed for each of the test instances in the removed fold, and the overall procedure was repeated until exhaustion of all five folds. With ∆ξ_k_ (**x**) denoting the differential log-hazard-ratio function for expression vector **x** for the model trained with the *k*-th fold removed, the cross-validation thus generates a collection of biomarker values for all *n* = 209 instances,

8 $$ C=\left\{\Delta {\xi}_{k_i}\left({\mathbf{x}}_i\right),i=1,\dots, n\right\}, $$

where *k*_*i*_ refers to the fold in which the i-th sample resides and **x**_i_ to its expression vector. Biomarker performance was then estimated using all *n* values of ∆ξ pooled together, as if they had been generated by a single model on a completely independent test set with *n* samples, an approach corresponding to the concept of “pre-validation” [32].

### Biomarker performance: the hazard ratio receiver operating characteristic

**Fig. 3:**
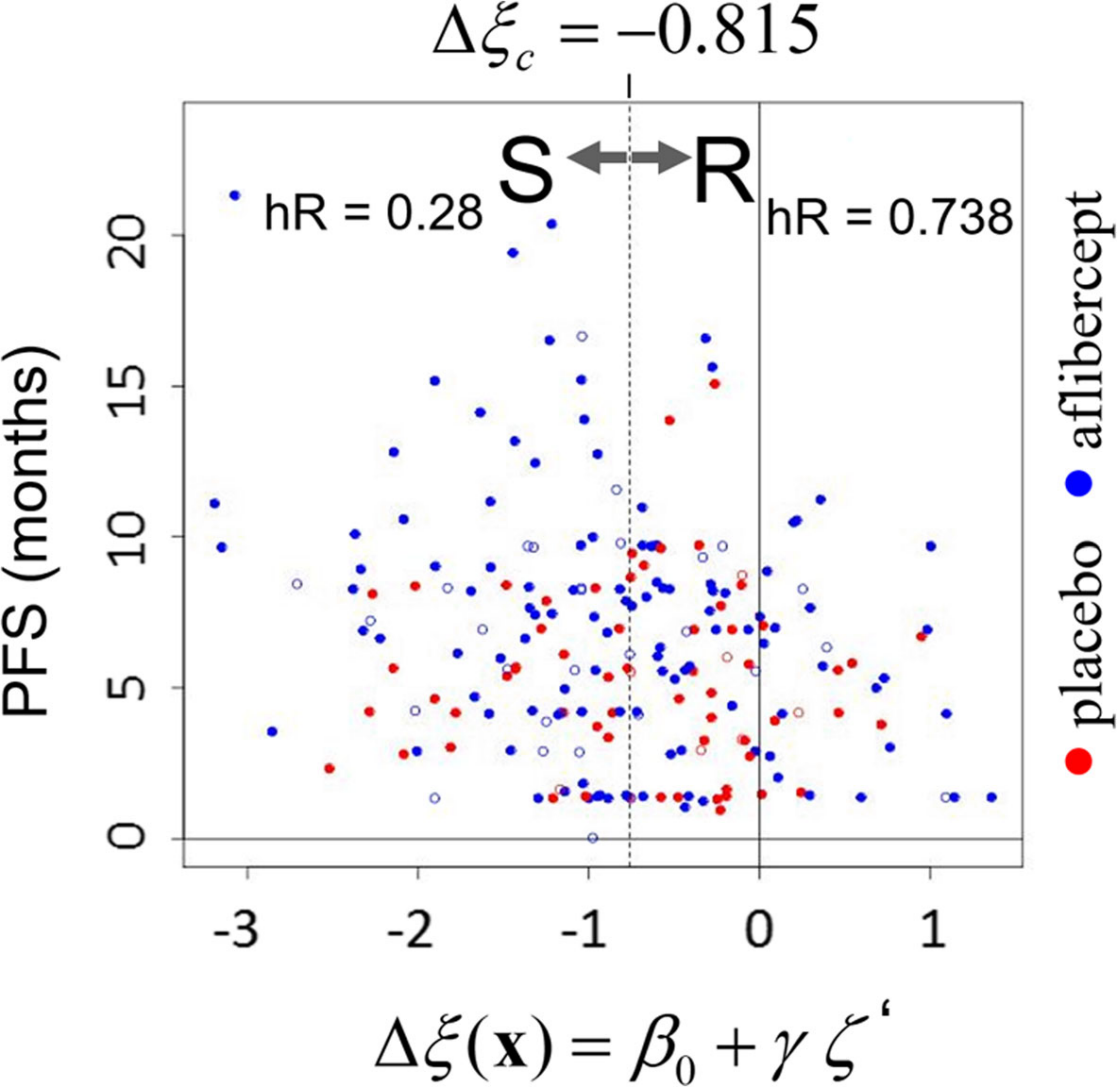
A survival scatter plot shows that ∆ξ is a predictor of differential patient sensitivity. For the subC-LP signature, observed PFS times are plotted versus the cross-validated differential log-hazard-ratio ∆ξ for each the *n* = 209 patients considered in the AFLAME panel. Points for control and aflibercept arm patients are displayed in red and blue, respectively,with a mix of censored (full dots) and uncensored (open dots) data in each category. The binary classification into “sensitive” (S) and “relatively-resistant” (R) groups that obtains with threshold ∆ξ_c_ = − 0.815 (dashed vertical line) is indicated by the arrows at the top of the plot, with resulting aflibercept-to-control hazard ratios hR shown alongside. For ∆ξ ≤ − 0.815, it can be seen that patients in the treatment arm (blue) tend to have longer survival than those in the control arm (red), with many blue dots higher than red dots, and that this asymmetry is greater than for patients with ∆ξ > − 0.815

A “survival scatter plot” (SSP) of observed survival time versus the cross-validated differential log-hazard-ratio ∆ξ can be used to gauge how well the model predicts differences in survival of the patients between the two treatment arms. An example is shown in Fig. 3, where the progression free survival time (PFS) is plotted against ∆ξ for the subC-LP signature. In the graph, each dot corresponds to a patient, with red and blue dots indicating individuals in the control and aflibercept arms, respectively (censored data is indicated by open circles, uncensored data by filled circles). Because the values of ∆ξ are derived from 5-fold cross-validation, test and training data used in prediction for each individual are thus independent, and Fig. 3 should reasonably reflect how well the model will generalize on similar types of data.

If the model illustrated in Fig. 3 is truly predictive of differential outcome, patients with ∆ξ < 0 should generally have markedly better survival in the aflibercept treatment arm than in the control arm, and conversely for those with ∆ξ > 0. These assertions are qualitatively verified, at least for large |∆ξ|: thus for patients with ∆ξ ≤ − 0.815 (a split at approximately the median value of ∆ξ, defining the left-hand side of Fig. 3) a fraction of the blue dots lies well above the red dots in the figure, indicating longer survival for the aflibercept-treated patients, with observed aflibercept to placebo arm hazard ratio hR = 0.28 for this group. For patients in the complementary range ∆ξ > − 0.815 (right-hand side of Fig. 3) the two populations of dots are too intermingled for easy visual discrimination, but the computed hazard ratio is hR = 0.738, still less than 1 and hence consistent with predicted ∆ξ being negative for the most of the patients in this group. The overall distribution of values of ∆ξ is thus consistent with the ‘moderate interaction’ scenario shown in Fig. 2c.

To go beyond the qualitative appraisal of Fig. 3, we quantify the correlation between survival times and ∆ξ by choosing a hard threshold ∆ξ = ∆ξ_c_, which splits the patient population into predicted aflibercept-sensitive (S) (∆ξ ≤ ∆ξ_c_) and aflibercept relatively-resistant (R) (∆ξ > ∆ξ_c_) response groups (see arrows pointing to the selected groups in Fig. 3). Within each group, the patients in the two treatment arms are then compared using a univariate Cox model (aflibercept relative to control), resulting in hazard ratios hR(S) and hR(R) and associated *P*-values pR(S) and pR(R), for S and R groups respectively (note that with the given order of treatment arm comparison, hR < 1 always indicates better survival in the aflibercept arm, whatever the response group).

**Fig. 4:**
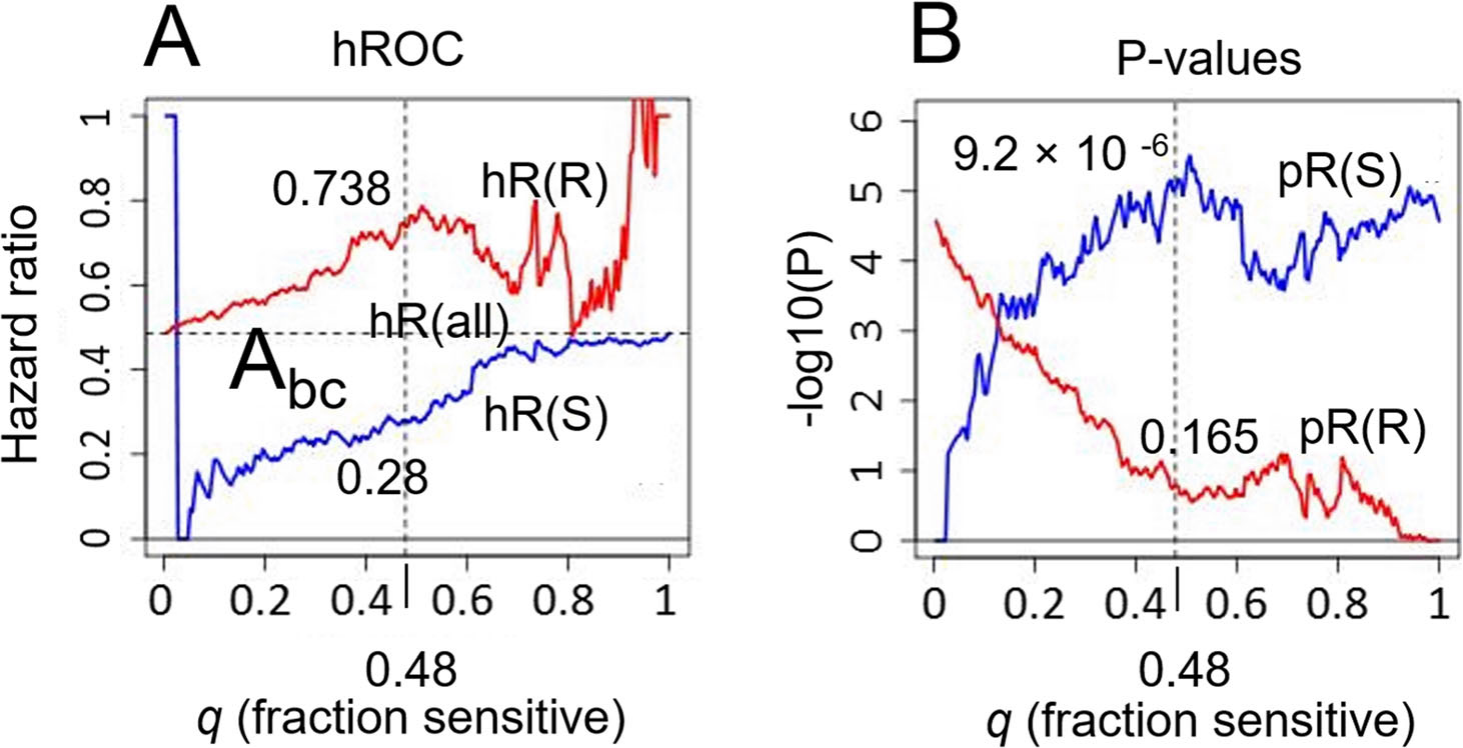
The hazard ratio receiver operating characteristic (hROC) quantifies the predictive performance of the biomarker. **a** For the subC-LP signature, the hazard ratios of aflibercept-treated patients versus baseline-treated patients in the relatively-resistant and sensitive populations, hR(R) (red) and hR(S) (blue) respectively, are plotted versus the fraction *q* of patients declared sensitive, all quantities being generated parametrically by sweeping the decision threshold ∆ξ_c_ left-to-right across Fig. 3. The horizontal dashed line labeled hR (all) denotes the aflibercept-to-control hazard ratio for all patients taken together (hR (all) = 0.486). The area A_bc_ between the red and blue curves is a global measure of the predictive performance of the signature. In this case A_bc_ = 0.3341. **b** Corresponding profile of *P*-values for hR(S) and hR(R). The vertical dashed line corresponds to decision threshold ∆ξ_c_ = − 0.815, for which hR(S) = 0.28, pR(S) = 9.2 × 10^− 6^, hR(R) = 0.738 and pR(R) = 0.165

The value of the threshold ∆ξ_c_ is so far arbitrary, and in fact we are free to compute hR(R) and hR(S) for all values of ∆ξ_c_, as the threshold is swept left to right across the x axis of Fig. 3, this procedure generating n + 1 discrete values of hazard ratios for each of the two patient groups, corresponding to n + 1 distinct binary partitions of the patient population. For each value of ∆ξ_c_ we can simultaneously record *q*, the fraction of individuals in the aflibercept-sensitive group (0 ≤ *q* ≤ 1). We can then parametrically express hR(R) and hR(S) as functions of *q*, the result being the “hazard ratio receiver operating characteristic” (hROC), which measures the tradeoff between stringency of patient selection and aflibercept-to-control treatment benefit for each of the patients groups. The hROC can be considered an extension of the so-called subpopulation treatment effect pattern plot or STEPP [16], with added emphasis on the separation between S and R groups as a function of decision threshold and concomitant tradeoffs. The hROC curves corresponding to Fig. 3 are shown in Fig. 4a, with red and blue lines denoting hR(R) and hR(S), respectively, both plotted against *q*. The concomitant profiles of *p*-values pR(R) and pR(S) are shown in Fig. 4b.

Note that a general property of the hROC is that if hR (all) denotes the hazard ratio between treatment arms for all patients taken together (horizontal dashed line in Fig. 4a at height hR = hR (all) = 0.486), then as *q* → 1 we have hR(S) → hR (all) (since in this limit the sensitive group consists of all the patients; see behavior of the blue line at the right of Fig. 4a), while hR(R) displays a large variance (as it is derived from a vanishingly small numbers of individuals; see the red line at the right of Fig. 4a). A similar behavior obtains for the opposite limit *q* → 0, but now with the roles of hR(S) and hR(R) reversed (left-hand side of Fig. 4a). In between these limits, the individual hazard ratio curves can vary; however, in the specific example of Fig. 4a the curves are well-separated, with hR(S) < hR(R) almost everywhere.

For a given threshold ∆ξ_c_ we can quantify the statistical significance of the cross- validated predictions by the *P*-value pR(S) [14]. Thus for ∆ξ_c_ = − 0.815 we have pR(S) = 9.2  10^− 6^ (vertical dashed line at q = 0.48, Fig. 4b) corresponding to the small hazard ratio hR(S) = 0.28 (Fig. 4a).

### Model optimization: the area between the curves

If a regression model is a good predictor of differential survival, then in general the corresponding hROC curves will be well-separated, ideally with hR(R) » hR(S) for a significant range of *q*, a situation which offers the possibility of a large treatment benefit for the sensitive group relative to the relatively-resistant group, combined with flexibility in setting a selection threshold. To give a more quantitative measure of the separation between the hROC curves, in a way which accounts for both height and width of the separating gap (Fig. 4a), we can compute the “area between the curves” *A*_*bc*_, defined by the expression

9 $$ {A}_{bc}={A}_{h_R}-{A}_{h_s}, $$

where ${A}_{h_R}$ and *A*_*hS*_ are the areas under the individual curves for hR(R) and hR(S), respectively, after a symmetrizing transformation of the hazard ratios resulting in $-1\le {A}_{h_{R, SR}}\le 1$ (Appendix A, Additional file 9). From Eq.(9) it can be seen that $\left|{A}_{bc}\left|\le \right|{A}_{h_R}\left|+\right|{A}_{h_s}\left|\le \max \right|{A}_{h_R}\left|+\max \right|{A}_{h_s}\right|=2\kern0.36em ,$ but because ${A}_{h_R}$ and *A*_*hS*_ are not independent, in practice we have |*A*_*bc*_ | ≤ 1.5 (Appendix A). Positive values of *A*_*bc*_ indicate predictive power of the biomarker which is consistent with the definition of the R and S patient groups, and better predictors will have a larger *A*_*bc*_. For the hROC shown in Fig. 4a, *A*_*bc*_ = 0.3341.

### Model optimization: choice of a decision threshold for patient stratification

Once a globally optimal model has been chosen (say on the basis of maximizing *A*_*bc*_), the decision threshold ∆ξ_c_ must be fixed so as to generate the actual patient assignments to sensitive (S) and relatively-resistant (R) response groups. This selection might be done in an ad hoc fashion by using visual inspection of the hROC curves to establish a thresholding “sweet spot”, for which the aflibercept treatment benefit for the sensitive group is thought adequate (e.g. by requiring hR(S) ≤ 1/3), but at a threshold ∆ξ_c_ that is not so stringent that the sensitive group is too small according to some pre-set limit (e.g. the sensitive group might be required to contain at least q = 1/4 of the total population of patients). A more principled approach is to use an objective function that quantitatively weighs in these considerations, by mathematically combining treatment cost/benefits for both groups with the sizes of the affected groups: this is done for the TNBC use case presented below (see Eq.(15)). In the Discussion section we also list some of the major constraints on the choice of the decision threshold.

However, for simplicity and continuity in the present discussion we considered just the fixed value of ∆ξ_c_ = − 0.815, indicated by the vertical dashed lines in Figs. 3 and 4a. This empirical threshold corresponds to a predicted upper bound on the aflibercept-to-control hazard ratio of exp.(− 0.815) = 0.4426 for the sensitive group, and can be seen to generate a reasonable partition of the patients: the resulting sensitive and relatively-resistant groups contain 100 and 109 patients, respectively (*q* = 100/209 = 0.48), and the assignments result in hazard ratios and *P*-values hR(S) = 0.28, pR(S) = 9.2 × 10^− 6^, and hR(R) = 0.738, pR(R) = 0.165. In other words, under the stratification induced by the threshold ∆ξ_c_ = − 0.815, about 1/2 of the patients are declared sensitive, and predicted to benefit from an almost four-fold reduction in hazard under aflibercept treatment relative to control, while the remaining 1/2 of the patients are declared relatively-resistant, and are predicted to experience considerably less (and here in fact statistically nonsignificant) benefit from aflibercept treatment relative to control.

### Resampling establishes model robustness and provides confidence intervals for model performance

The results described in connection with Figs. 3 and 4 were obtained from a single 5-fold cross validation of the subC-LP signature conducted on the entire AFLAME data set of *n* = 209 samples. To gauge the robustness of these results under generalization, we used bootstrap resampling [33] to extend these point-wise observations, and establish distributions and confidence intervals for hR(S), hR(R) and the allied performance metrics such as *A*_*bc*_. Note that the bootstrap resampling procedure simulates as much as possible real-world variation in both training and test sets, and hence helps anticipate the variation in predictive performance to be expected when the signature is applied to a completely new data corpus.

To implement bootstrap resampling, the 5-fold cross-validation procedure was embedded in an outer computational loop, in which 1000 random resamplings with replacement of the *n* = 209 samples were generated, with a full cross-validation done on every resampled data set. For each resampled realization, the five folds required for cross-validation were also (randomly) re-generated from scratch. Patient classification into the two response groups was done for each resampling, with fixed decision threshold ∆ξ_c_ = − 0.815, and the resulting bootstrapped hazard ratio values hR(S)^∗^ and hR(R)^∗^ and other quantities were recorded under each resampling. Following completion of the outer resampling loop, statistical analyses were conducted on the collected data to generate confidence intervals and distributional plots for the quantities of interest.

**Fig. 5:**
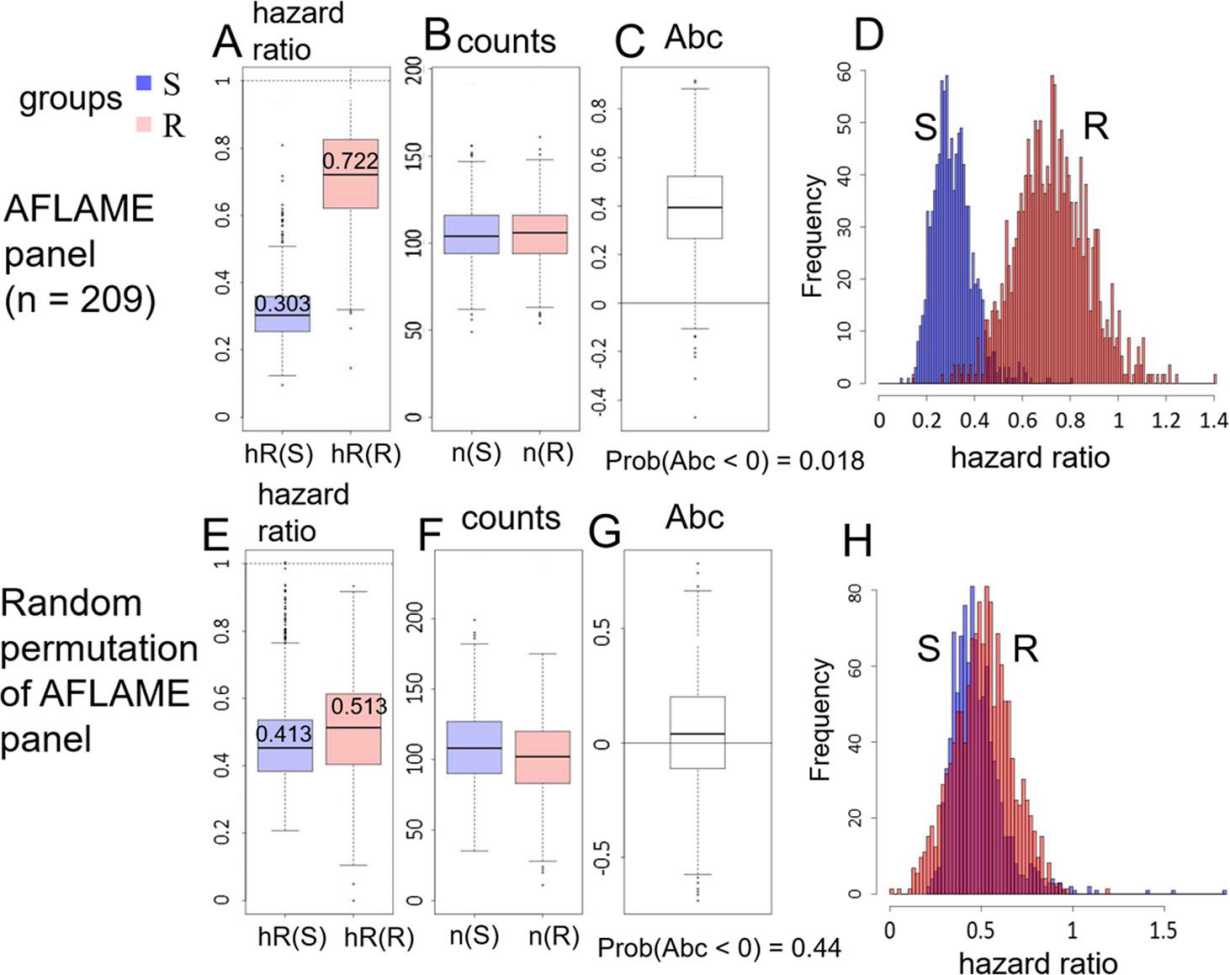
Bootstrap resampling provides confidence intervals for anticipated performance on independent CRC data sets. Bootstrap resampling of 5-fold cross-validation of the subC-LP signature was used to generate distributions of hazard ratios for sensitive and relatively-resistant groups (**a**), number of patients in each group (**b**), and area between curves Abc (**c**). Predictive risk for the signature is found to be pRisk = P (Abc < 0) = 0.018. The histograms in (**d**) show in more detail that the distributions of hR(S) and hR(R) barely overlap. Figures **e**-**h** display the corresponding statistics generated from a randomized data set, showing that the distributions of hR(S) and hR(R) (**e**, **h**) are almost completely overlapping, as expected for this negative control. The predictive risk for the randomized model is 0.44 (**g**)

Results of bootstrap resampling for the subC-LP signature are shown in Fig. 5. Thus, side-by-side box plots for the hazard ratios (Fig. 5a) show that over the distribution, the hazard ratios for the sensitive group (blue) are almost always smaller than for the relatively-resistant group (red). The median values and 95% confidence intervals for the hazard ratios are given by hR(S) = 0.303 [018, 0.50]_0.95_, hR(R) = 0.722 [0.45, 1.04]_0.95_, and the corresponding histograms (Fig. 5d) confirm in detail that the distributions for hR(S) and hR(R) barely overlap. Distributions of the number of patients n(S) and n(R) assigned to the respective response groups are shown in Fig. 5b: the median values are seen to be nearly equal (median n(S) = 104, median n(R) = 105), indicating that the fixed decision threshold ∆ξ_c_ = − 0.815 generally split the patient population in two. Furthermore, the resampled values of hR(S) and n(S) are not correlated (data not shown), so that there is no necessary ‘penalty’, in terms of a small value of n(S), for realizations with otherwise desirably small hR(S).

A box plot for the area between curves *A*_*bc*_ (Fig. 5c) shows that under the bootstrap resampling *A*_*bc*_ is almost always positive. While *A*_*bc*_ > 0 indicates prediction consistent with observed PFS outcome (that is, hR(R) > hR(S) for most of the range of the hROC, as in Fig. 4a), conversely *A*_*bc*_ < 0 indicates an inconsistent or ‘failed’ prediction by the signature (that is, hR(R) < hR(S) for most of the range of the hROC). The ‘predictive risk’

10 $$ pRisk=\Pr \left({A}_{bc}<0\right) $$

is thus the estimated probability of failure of the signature under generalization to arbitrary test sets. A small predictive risk corresponds to a signature for which we have high confidence in predictive success, and thus pRisk << 1 can be considered to have the same validation status as a small *P*-value. For the subC-LP signature (Fig. 5c) pRisk = 0.018 < < 1, indicating a high confidence predictor.

As a negative control, we also performed bootstrap resampling of the subC-LP signature on a single, randomized re-assignment of the gene expression profiles. To that effect, the *n* = 209 gene expression profiles were randomly permuted once, with respect to all clinical outcome labels, thereby breaking any potential correlation between gene expression and PFS. The resulting distributions of hR(S) and hR(R) (Fig. 5e and h) are seen to be almost completely overlapping, and the predictive risk, derived from the distribution of *A*_*bc*_ (Fig. 5g), is almost ½ (pRisk = 0.44). In summary, when trained on a randomized data set the subC-LP signature simply generates random, undifferentiated predictive outcomes (with nearly 50–50 ‘coin-flip’ probabilities), as expected.

Finally, we can use the distribution of *A*_*bc*_ from the randomized model (Fig. 5g) to define a null hypothesis. A *P*-value for prediction can then be computed from the one-sided test

11 $$ P=\Pr \left({A}_{bc}>{A}_{bc}^{obs}\right)\kern0.36em , $$

where the ‘observed’ value ${A}_{bc}^{obs}=0.3441$ is obtained from the non-randomized, non-bootstrapped model (Fig. 4a). From Eq. (11) we find *P* = 0.019, consistent with the small pRisk = 0.018 obtained above.

### A comparison of resampling results indicates good predictive performance for both subC-LP and subC-CMS signatures

For comparison purposes, the resampling analysis described above was extended to a number of other signatures. Foremost was the subC-CMS signature, based on the centroids for the CMS subtype classification [18] (with AFLAME-fitted Cox coefficients given in Table 1). Additionally, a signature called subC-PAM50, based on the breast cancer-relevant PAM50 subtype classification [34, 35], was considered. This signature was expected to be a negative control, on the assumption that breast cancer subtypes should not be relevant to prediction in colorectal cancer. Finally, a signature designated subC-RANDOM was constructed as a true negative control, using five randomly chosen centroids, defined on a set of 50 randomly selected genes, with random components in all five centroid vectors.

**Fig. 6:**
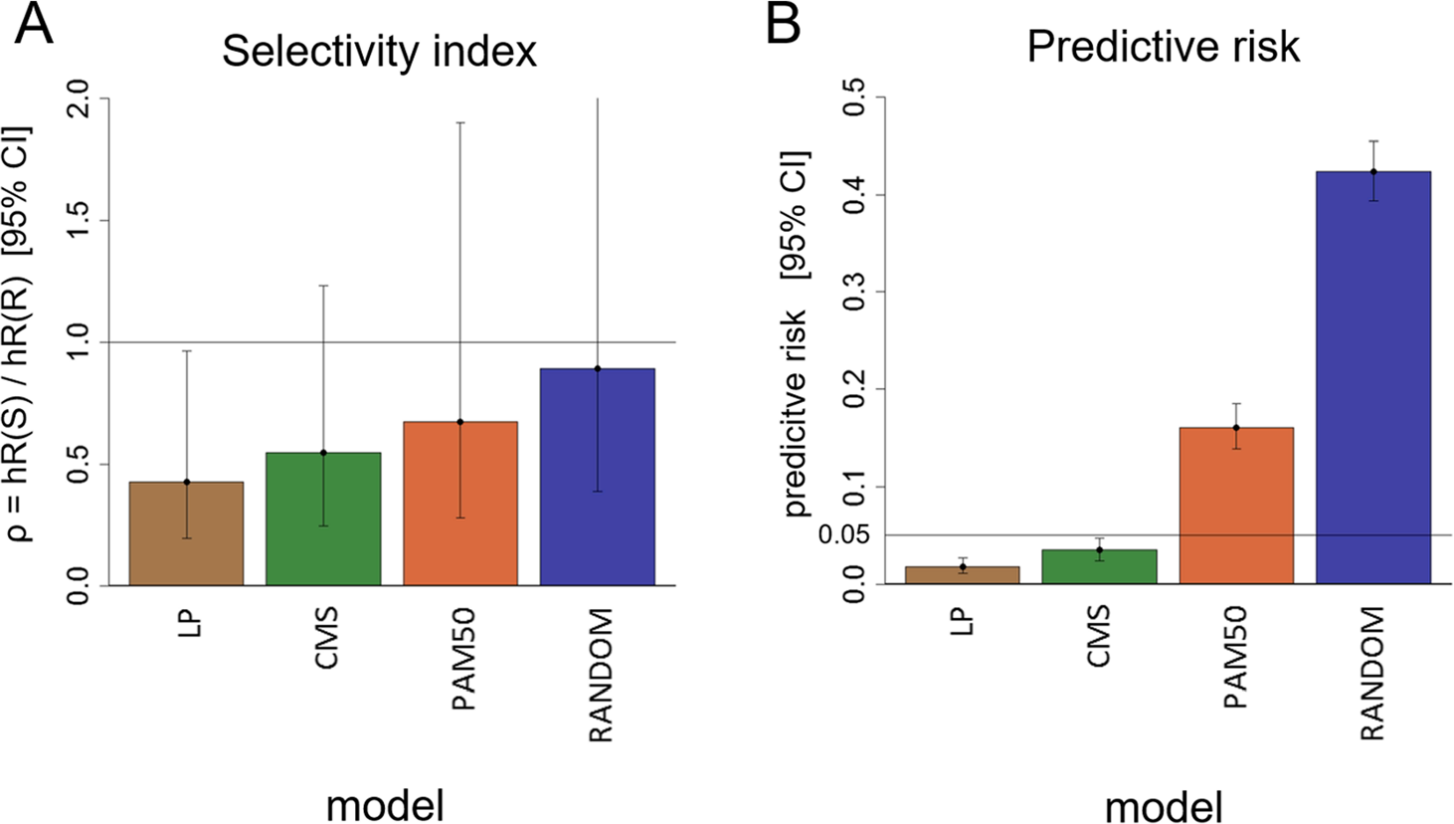
Resampling analysis predicts robust performance for subC-LP and subC-CMS signatures. Systematic bootstrap resampling or permutation tests were performed on four signatures (LP, CMS, PAM50, RANDOM). **a** Bar plot of median and 95% confidence intervals for the specificity index *ρ* = *hR*(*S*)/*hR*(*R*). **b** Corresponding values for the predictive risk. Bootstrap resampling for the LP or CMS signatures indicate good predicted performance. Resampling for the negative controls PAM50 or RANDOM generate statistically non-significant results

Figure 6 summarizes results for the four subC signatures (LP, CMS, PAM50 and RANDOM), examined under resampling with fixed decision threshold ∆ξ_c_ = − 0.815. Focus was on the ‘selectivity index’ defined by

12 $$ \rho =\frac{hR(S)}{hR(R)}, $$

where ρ < < 1 is indicative of high selectivity, and ρ ~ 1 indicative of no selectivity at all. Full bootstrap resampling for subC-LP (Fig. 6a) resulted in ρ = 0.426 [0.19, 0.96]_0.95_, with predictive risk = 0.018 (Fig. 6b), as already noted. The LP signature thus generates a statistically significant prediction, and reasonable performance, with a median difference in hazard ratios hR(S) and hR(R) of more than 2-fold. In comparison, full bootstrap on the subC-CMS signature (Fig. 6a and b) results in the estimate ρ = 0.547 [0.25, 1.23]_0.95_, with predictive risk = 0.035. Prediction by the subC-CMS signature is thus also statistically significant, but with performance not quite as good as for the subC-LP signature.

For the negative controls, full bootstrap on the PAM50 signature (Fig. 6a and b) generates ρ = 0.675 [0.28, 1.9]_0.95_, with predictive risk = 0.16, so that performance of the PAM50 signature is not statistically significant (although a predictive trend might still be indicated). Finally, full bootstrap on subC-RANDOM (Fig. 6a and b) generates ρ = 0.89 [0.39, 2.6]_0.95_, with predictive risk = 0.42, so that as, expected performance, of the RANDOM signature is not statistically significant, with median selectivity index close to 1.

The main performance results for the LP, CMS, PAM50 and RANDOM signatures are shown in Table 2.

**Table 2:** Summary of bootstrap resampling results for the four subC signatures compared in the study

	subC signature:			
Quantity:	LP	CMS	PAM50	RANDOM
hR(S)	0.303 [0.18, 0.50] _0.95_	0.35 [0.22, 0.56] _0.95_	0.368 [0.20, 0.78] _0.95_	0.443 [0.27, 0.82] _0.95_
hR(R)	0.722 [0.45, 1.04] _0.95_	0.65 [0.40, 0.94] _0.95_	0.526 [0.35, 0.79] _0.95_	0.519 [0.21, 0.78] _0.95_
ρ = hR(S) / hR(R)	0.426 [0.19, 0.96] _0.95_	0.547 [0.25, 1.23] _0.95_	0.675 [0.28, 1.9] _0.95_	0.89 [0.39, 2.6] _0.95_
n(S)	104 [72, 139] _0.95_	108 [59, 151] _0.95_	74 [29, 119] _0.95_	100 [48, 158] _0.95_
pRisk	0.018	0.035	0.161	0.424

### Analysis of the response groups: patient selection matrix and Kaplan-Meier (KM) plots

**Fig. 7:**
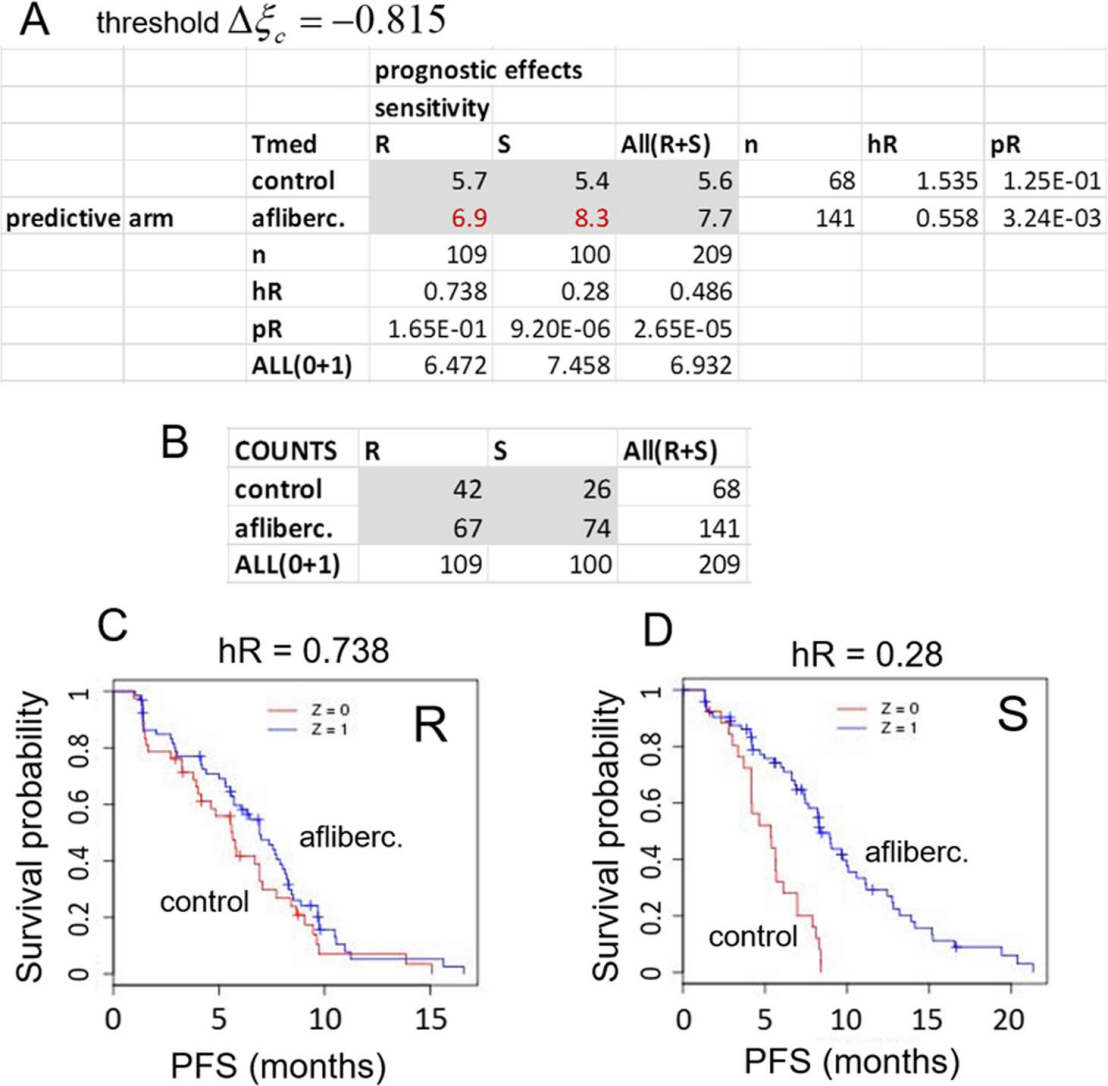
The Patient Selection Matrix is a summary of prognostic and predictive powers of the signature. **a** The Patient Selection Matrix (PSM) (shown for the 5-fold cross-validated subC-LP signature) is built around the 2 × 2 contingency table of outcomes according to {treatment arm × response group} classification of the patients (here for decision threshold ∆ξ_c_ = − 0.815). The cells in the core of the table (grey) contain median survival times (months) for each category of the classification. Margins labeled n, hR and pR indicate the total number of subjects, hazard ratio, and attendant *P*-value, respectively, arising from differential survival analysis along the corresponding rows or columns. **b** Number of patients in each {treatment arm × response group} category. **c** KM plot for the *n* = 109 patients in the relatively-resistant group and **d**, KM plot for the *n* = 100 patients in the sensitive group

The consequences of patient classification into discrete, biomarker-dependent groups by a given signature can be explored in more detail by using a “patient selection matrix” (PSM) which is built around a 2 × 2 contingency table of patient outcomes according to {treatment arm × response group} combinations. The PSM resulting from 5-fold cross-validation (without resampling) of the subC-LP signature with decision threshold ∆ξ_c_ = − 0.815 is shown in Fig. 7a. The corresponding hROC is shown in Fig. 4a. Median survival times for each combination of factors (including those resulting from All ≡ R + S grouped together), are displayed in the six central cells of the table (grey area), the surrounding column and row margins indicating in outward succession the total number of patients, hazard ratios and *P*-values for two-group comparisons along the corresponding axes. Reading the PSM horizontally (i.e. along each of the rows in Fig. 7a labeled control or aflibercept), one is looking at outcomes *within* each treatment arm separately, so that the prognostic power of the signature is in focus. Reading the PSM vertically (i.e. along the each of the columns in Fig. 7a labelled R or S), one is looking at effects *between* treatment arms in each response group separately, so that in this case, the predictive power of the signature is examined.

Inspection along the columns of Fig. 7a for predictive effects thus shows that the relatively-resistant group R (*n* = 109) exhibits a statistically non-significant aflibercept-to-control hazard ratio hR = 0.738 (pR = 0.165), while the sensitive group S (*n* = 100), exhibits a statistically significant hazard ratio hR = 0.28 (pR = 9.2 × 10^− 6^). The corresponding survival curves (Kaplan-Meier or “KM plots”) are shown in Fig. 7c and d. The corresponding gains in median PFS time, aflibercept relative to control arm, can be read from the table by direct subtraction and are found to be ΔPFS = 1.2 and 2.9 months for R and S groups, respectively.

Inspection along the rows of Fig. 7a for prognostic effects, comparing sensitive versus relatively-resistant groups within each treatment arm, shows that the control arm (label 0) exhibits a statistically non-significant hazard ratio (hR = 1.53, pR = 0.125, but with perhaps a trend toward hR > 1). On the other hand, the aflibercept arm (label 1) exhibits a significant hazard ratio hR = 0.558 (pR = 3.2 × 10^− 3^), indicating that the signature is indeed prognostic in that treatment arm.

Taken together, these results show that the predictive power of the subC-LP signature comes from combination of a strong positive prognostic effect in the aflibercept arm, with either a non-existent, or a weaker and negative prognostic effect in the placebo arm.

Finally the PSM for the more stringent selection threshold ∆ξ_c_ = − 1.5 (corresponding to a hazard ratio threshold = 0.223) is shown in Supplementary Figure 1. This threshold selects for a much smaller sensitive group (37 patients, 18% of total), but one with hR(S) = 0.168, while that of the relatively-resistant group (172 patients, 82% of total) is hR(R) = 0.564. The corresponding gains in median PFS time are found to be ΔPFS = 1.8 and 4.8 months for R and S groups, respectively, to be compared with ΔPFS = 1.2 and 2.9 months, respectively, obtained above with the less stringent ∆ξ_c_ = − 0.815.

### A signature for triple-negative breast cancer predicts the existence of sensitive and resistant subgroups of patients

As an additional application of the general methodology presented above, we considered treatment of triple-negative breast cancer (TNBC) with the small molecule iniparib [12, 13]. The gene expression data analyzed here was generated by microarray profiling (Affymetrix HuGene1.0ST microarray) of FFPE samples from phase 2 and phase 3 two-arm studies conducted to test the efficacy of iniparib in combination with standard-of-care chemotherapy in patients with metastatic recurrence of TNBC [12, 13]. In each of the trials, patients were randomly assigned to one of two treatment arms, one using standard-of-care cytotoxic gemcitabine/carboplatin combination therapy alone (the “control” arm), and the other with the same cytotoxic treatment augmented by iniparib (the “iniparib” arm). For the analyses which follow, we focused on a subset of the data consisting of *n* = 210 gene expression profiles obtained after quality-control of samples for tumor content, confirmation of negative hormone receptor status, and quality of microarray hybridization. Data was batch-corrected, quantile normalized, log2-transformed and standardized in accordance with Eq.(1). For all patients taken together, a significant treatment benefit in progression free survival (PFS) time from iniparib relative to control was observed (*P*-value *P* = 1*.*4 *×* 10^*−* 2^, hazard ratio *hR* = 0*.*673 [0.49, 0.92]_95%_). We wished to establish whether the patients could be further stratified into “sensitive” and “resistant” groups.

### Two alternative regularized multivariate cox models can be used to generate predictive signatures

As in the case of CRC, because of the high dimensionality of the gene expression data, it was essential that the models be appropriately regularized [27] through feature selection and/or transformation of selected features. Because subtypes of TNBC alone have not been well characterized, we could not apply the subC method described above for CRC. Among many possible alternatives [36–39], we focused instead on two specific methods to generate the reduced-dimensionality covariates *x*˜_*l*_, *l* = 1*, . . . , K* of Eq.(2):

1. Mechanism of action (MOA) model: in this approach the gene expression data matrix was from the start restricted to a collection of genes representative of the mechanism of action of iniparib, which is presumed to induce oxidative stress in target cells through inhibition of the enzymes thioredoxin reductase 1 and 2 (Zachayus JL et al: Iniparib is a Cytotoxic Anti-Tumor Prodrug Bioactivated by TrxR1/2. Submitted for publication). The collection of 101 genes (82 of which were represented on the microarrays used in the profiling) consisted primarily of genes involved in the oxidative stress response pathway (Additional file 8). The initial selection, based on a priori knowledge, thus reduced the dimensionality of the data matrix from *p* = 20,756 to *p*^*’*^ = 82. Feature selection using ranking of genes by their interaction *p*-value derived from univariate gene-by-gene Cox models of PFS was then applied to further reduce the number of selected genes to a value *mtop*, where *mtop* (1 ≤ *mtop* ≤ p’) is a tuning parameter of the model. The selected genes were then directly used as covariates in a K = 1 principal components model.
2. Supervised principal components (SPC) model: here, supervised principal components [36] analysis was used to generate the Cox model. Starting with the full normalized and standardized *n* × *p* data matrix **X**, univariate feature selection was first directly applied to reduce the number of genes to *mtop*, where as in the MOA model, *mtop* (1 ≤ *mtop* ≤ *p*) is a tuning parameter. This step resulted in an *n* × *mtop* data matrix **Y**. Dimensionality was then further reduced by defining the variables *x*˜_*l*_, *l* = 1*,*. *.*.*, K*, to be the projections of the individual gene expression vectors **x** in **Y** onto the first *K* principal components of **Y**. Formally,

13 $$ \tilde{x}_{l}\kern0.5em ={\mathbf{u}}_l^T\cdot \mathbf{x},\kern0.5em l=1,\dots, K $$

where **u**_*l*_ is the *l*-th principal component vector of **Y**. In what follows, *K* = 1 was used throughout, as cross-validation indicated that at given *mtop* this value was generally optimal for prediction.

It can be noted that the MOA and SPC models embody two complementary approaches for predictive signature discovery. The MOA model is a biased approach which exploits a priori knowledge of potentially relevant genes to maximize the probability of signature discovery in a dimensionally shallow data set (*p* ∼ *O*(100’s)), where the signal is presumably not masked by noise from many genes with false positive associations with outcome. However the MOA approach can fail if the set of genes considered a priori is simply inappropriate (we made the wrong guess) and does not contain the signature in the first place. On the other hand, the SPC approach starts with a much larger, unbiased data set (*p* ∼ *O*(10^4^)), in which the signature, if it exists, has certainly a better a priori chance of occurring than in any randomly chosen subset. However the SPC approach can also fail, if the number of samples (*n* ∼ *O*(100’s) typically) is insufficient to power the model enough, to overcome the much larger number of false positive associations inherent in such an unbiased approach.

### The area between curves is used to optimize feature selection

**Fig. 8:**
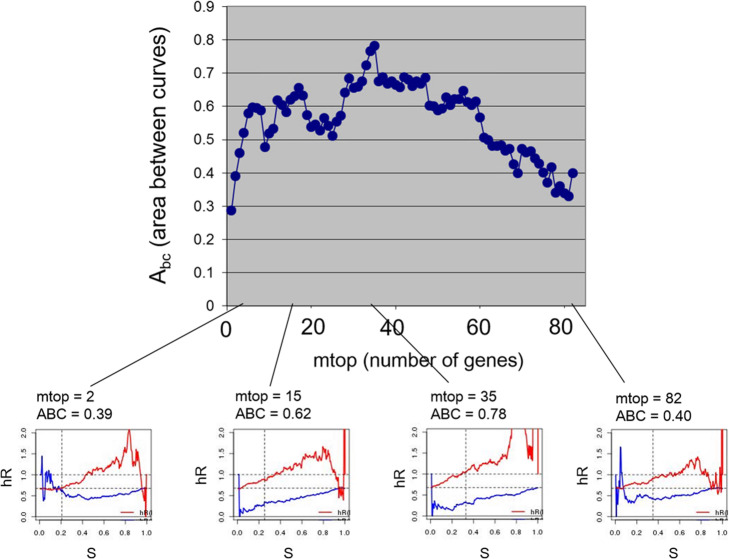
The area between hROC curves (Abc) is used for model optimization in prediction for TNBC. The area between the hazard-ratio ROC curves (Abc) is plotted as a function of mtop, the number of genes used by feature selection in the multivariate Cox MOA model for TNBC. Representative hROC curves for specific values of mtop are shown below. The hROC curves for mtop = 35 are the most separated, corresponding to the maximum value of Abc

As for a given model the area between the curves provides an overall figure of merit for all possible splits into sensitive and resistant groups on the basis of ∆*ξ*_*c*_, it can be used for model optimization. In Fig. 8*Abc* is plotted against the number *mtop* of genes selected in the MOA model, in the entire range 1 *≤ mtop ≤* 82. Models with very few genes (e.g. *mtop* = 1) or all the genes (*mtop* = 82) are clearly suboptimal, with *Abc ≈* 0*.*3 and *≈* 0*.*4, respectively. The largest value of *Abc* occurs for *mtop* = 35 (*Abc* = 0*.*78), which defines the optimal value of that parameter. The thumbnail plots of the individual hROCs at the bottom of Fig. 8 have been added to show how their appearance changes as a function of *mtop*. It can be visually appreciated that the hROC with *mtop* = 35 has the largest separation between hR(R) and hR(S) curves.

### The area between curves also enables selection between competing models

**Fig. 9:**
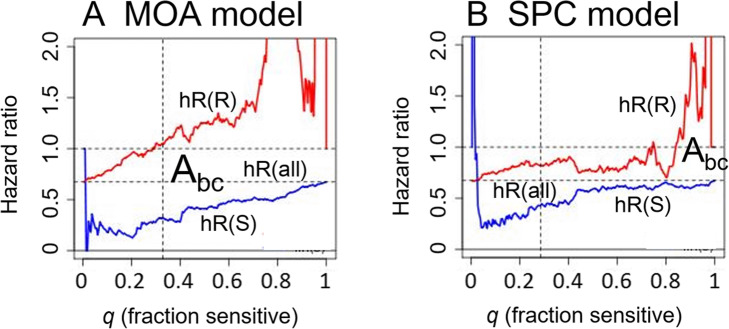
The area between curves is used to compare competing models in prediction for TNBC. hROCs are shown for the MOA (**a**) and SPC models for TNBC (**b**). The MOA model has superior predictive performance, with A_bc_ = 0.782 for the MOA versus A_bc_ = 0.378 for the SPC model

The values of *Abc* which result from individual model optimization can be used to compare the maximum predictive power of different models on the same data. Figure 9a and b show the hROCs which obtain from cross-validation of the optimized MOA and SPC models, respectively. The SPC model uses parameters *mtop* = 50 and *K* = 1, optimized using the same maximum *Abc* criterion as for the MOA model. While the cross-validated predictions of the SPC model are statistically significant (*pR*(*S*) = 6*.*4 *×* 10^*−*3^ for ∆*ξc* = *−*1), they result in an hROC with markedly smaller *Abc* than for the MOA model, with *Abc* = 0*.*3781 for SPC versus Abc = 0*.*7817 for MOA model. In what follows, we pursued analysis using the superior MOA model.

### An objective function can be used to optimize the decision threshold

**Fig. 10:**
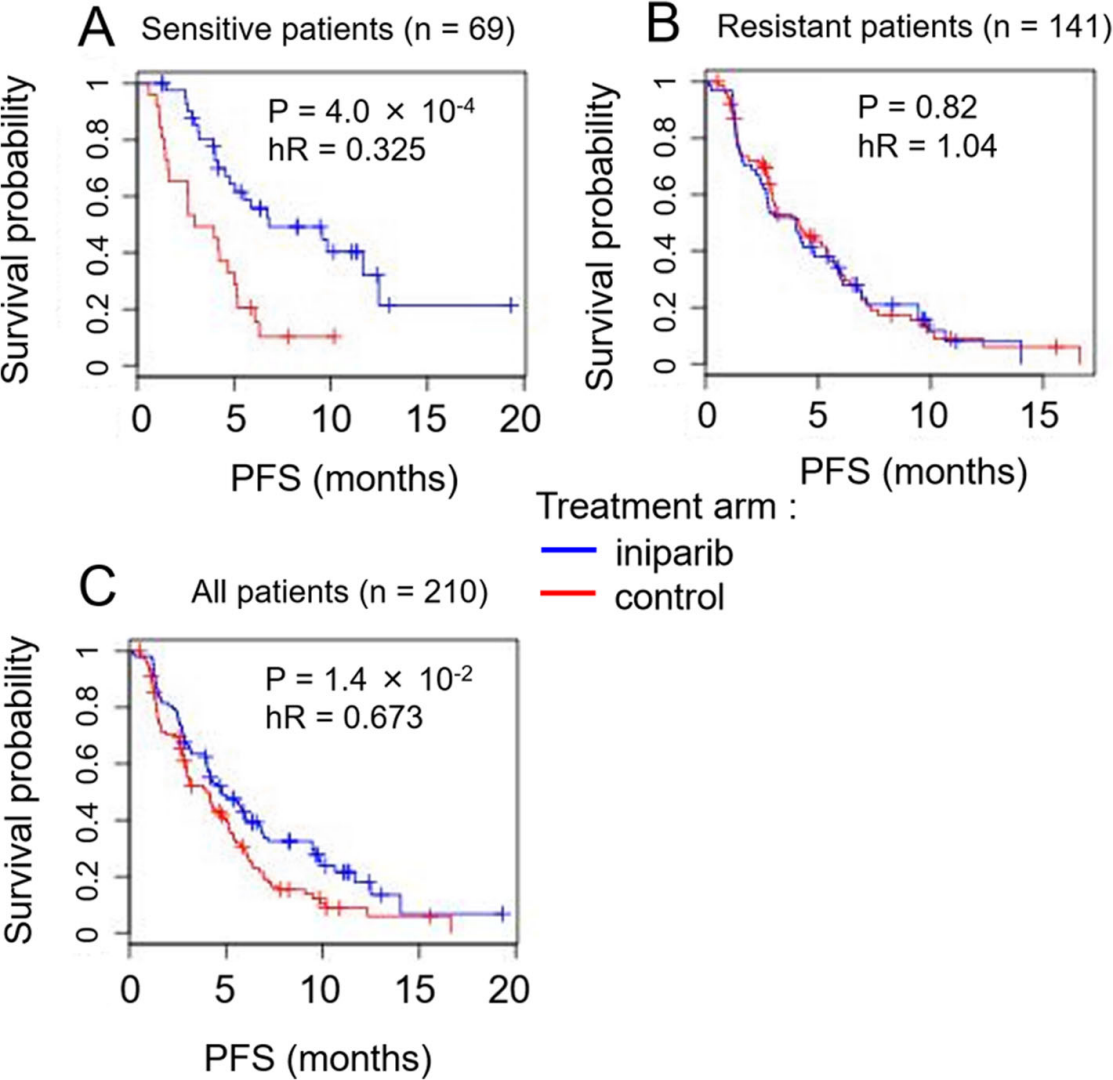
Survival plots for predicted sensitive-resistant groups in TNBC according to the MOA model and the ad hoc threshold Δξ_c_ = − **1.** Survival curves for the two treatment arms are compared for each patient group separately (in all cases red = control arm, blue = treatment arm). **a** Survival curves for the patients in the sensitive group, showing the large difference in survival between treatment arms. **b** Survival curves for the patients in the resistant group, showing nearly identical survival probabilities. **c** Survival plots for all patients (S + R groups), shown as reference

As in the case of the subC model applied to CRC, we first explored using an *ad hoc* decision threshold on the MOA model, choosing ∆*ξc* = *−*1, with split indicated by the vertical line in Fig. 9a. The resulting “sensitive” and “resistant” response groups contain 69 and 141 TNBC patients, respectively, and the assignments result in hazard ratios and *P*-values *hR*(*S*) = 0*.*325, *pR*(*S*) = 4 *×* 10^*−*4^, and *hR*(*R*) = 1*.*04, *pR*(*R*) = 0*.*82, respectively (Fig. 10). Thus, under the stratification induced by the threshold ∆*ξc* = *−*1, 1/3 of the patients are declared sensitive, and predicted to benefit from an almost three-fold reduction in hazard under inparib treatment relative to control, while the remaining 2/3 of the patients are declared resistant, and are predicted to experience little (statistically nonsignificant) benefit from inparib treatment relative to control.

While the *ad hoc* threshold ∆*ξc* = *−*1 gives a reasonable partition of the patient population, a more principled approach for setting ∆*ξc* is to rely on an objective function, which mathematically weighs costs and benefits for a given value of the threshold. The cost/benefit terms to enter the objective function depend on the ultimate use of the predictive signature, and will not be the same for a new clinical trial, where the aim is to maximize demonstrable treatment effects in a possibly small set of patients, as for routine clinical treatment, where the aim is to be as inclusive as possible.

**Fig. 11:**
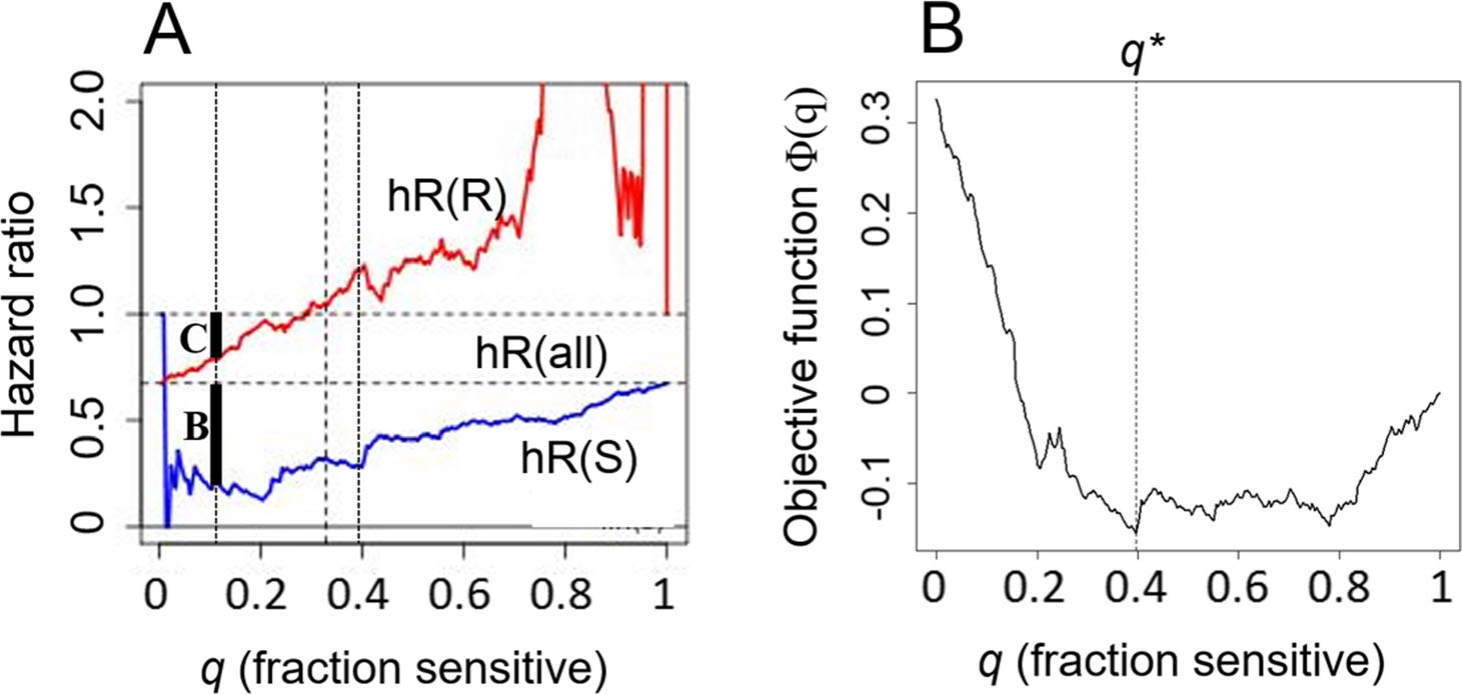
An objective function enables principled optimization of the signature threshold. **a** hROC for the MOA signature with mtop = 35, showing origin of factors discussed in connection with Eq.(16) in A. The short black bar labeled “C” refers to the treatment cost, and the bar labeled “B” to the treatment benefit terms entering into Eq.(14). **b** Optimization of the objective function φ(q) occurs for q∗ = 0.4 (vertical dashed line). The corresponding threshold is ∆ξ_c_ = − 0.73, with hazard ratios hR(S) = 0.28 and hR(R) = 1.22

Here we consider an objective function *φ* that might apply to routine clinical treatment, and which accounts for 1) the benefit to patients classified into the sensitive group, and treated with iniparib in addition to standard-of-care, and 2) the cost, through loss of treatment benefit, if any, to patients classified into the resistant group, and who were given standard-of-care treatment only. To capture these two terms, we chose a simple analytic form

14 $$ \varphi (q)=-q\cdotp \left(h0- hS(q)\right)+\left(1-q\right)\cdotp \max \left(1- hR(q),0\right) $$

where *q* is the fraction of patients in the sensitive group, (1 - *q*) the fraction of patients in the resistant group, with 0 *≤ q ≤* 1; where *h*_*S*_ (*q*) and *h*_*R*_(*q*) are the hazard ratios for the sensitive and resistant patient groups, respectively, and *h*_0_ is the hazard ratio for all patients (Fig. 11). Note that the overall sign of *φ*(*q*) is chosen such that it corresponds to a function to be *minimized* (i.e. it is indeed a cost function). In Eq.(14) the factor (*h*_0_ *− h*_*S*_ (*q*)) measures treatment *benefit* for the sensitive patients group (dark bar B in Fig. 11a). The factor max(1 *− hR*(*q*)*,* 0) on the other hand measures the (denial of) treatment *cost* to the patients in the resistant group (dark bar C in Fig. 11). Note that this second factor is gated-out for values of *q* for which *hR*(*q*) *>* 1. The two factors are weighted by the relative frequencies of sensitive and resistant patients, respectively.

The optimum patient split *q*^∗^ is found by minimization of the cost function,

15 $$ q\ast \kern0.5em =\mathrm{argmin}\left(\varphi (q)\right),\kern1em 0\le q\le 1 $$

from which the optimal threshold ∆*ξ*_*c*_^∗^ is also uniquely determined.

Optimization of Eq.(14) according to Eq.(15) (Fig. 11b) results in a partition of the patients with *q*^∗^ = 0*.*4, corresponding to ∆*ξ*_*c*_^∗^ = *−*0*.*73, and hazard ratios *hR*(*S*) = 0*.*28 and *hR*(*R*) = 1*.*22. Note that these values are close to those obtained with the *ad hoc* threshold ∆*ξc* = *−*1, for which *q* = 0*.*33, *hR*(*S*) = 0*.*33 and *hR*(*R*) = 1*.*05 (Fig. 9a), but reflect a more principled choice. Evidently, Eq.(15) can be modified to embody additional or different cost/benefit terms if required.

## Discussion

### Generality of the methodology

The approach for deriving predictive biomarkers was illustrated with two examples of two-arm clinical trials, concerning either CRC or TNBC patients. Specific models (subC, MOA, SPC) were initially used to reduce the dimensionality of the input data set. In each case however, the methodology presented here could then be applied to effect the construction of a predictive signature. The overall approach we have presented is thus quite general.

### Constraints on the choice of the decision threshold

The choice of the decision threshold Δξ_c_, which splits patients into the two groups termed resistant (R), and sensitive (S) is of great practical consequence. If we assume a scenario in which patients classified into the S group are treated with a given agent (e.g. aflibercept, iniparib), while those in the R group are not (i.e. they remain under the previous standard-of-care), optimization of the choice of Δξ_c_ is guided by a number of considerations:

1. we wish to see the treatment-to-control hazard ratio for the S group as small as possible, thereby maximizing their treatment benefit,
2. we wish to see a large difference in hazard ratio between R and S groups, thereby justifying the stratification in two groups,
3. we wish the S group to be not vanishingly small, so that at least some patients benefit from treatment, and so that in a clinical trial (as opposed to routine clinical setting) patient accrual times do not become prohibitively long,
4. in a routine clinical setting (as opposed to a clinical trial), we do not wish to deprive patients who might actually benefit from treatment, so that the hazard ratio for the R group should ideally be greater than or equal to 1.

The considerations listed above are constraining and strongly guide the choice of the decision threshold. In the TNBC use case presented above, we strove to incorporate them into a single mathematical objective function (Eq.(14)), and this resulted in a principled decision process for a routine clinical setting. However, an objective function is not strictly required in every case. For instance, in designing a new clinical trial for CRC (as opposed to planning for routine treatment), the main constraint is that the fraction of incoming patients declared sensitive cannot be too small, because otherwise patient accrual times will become prohibitively long. In practice, more than quadrupling accrual time might be considered inacceptable. This sets a lower bound of q = 0.25, which is achieved with Δξ_c_ = −1.32, for which the predicted hazard ratio of the selected patients is 0.2 (Fig. 4a). The total number of patients required to sufficiently power the resulting study can then be readily computed.

## Conclusions

A general approach for deriving a predictive, as opposed to prognostic, gene expression signature from two-arm clinical trials with concomitant gene expression profiling was presented. This general methodology was combined with more specific modeling steps. As initial steps in the modeling process, we considered for instance the subtype correlation (subC) model based on intrinsic molecular subtypes in CRC, or the mechanism of action (MOA) models, based on the known mechanistic pathways of the drug iniparib in TNBC. For CRC, the approach was applied to AFLAME, a two-arm clinical study for colorectal cancer involving the anti-angiogenic molecule aflibercept. Two related signatures, of similar predictive performance, were thus found, and under extensive cross-validation and resampling were shown to be robust, and hence are expected to be generalizable to independent CRC panels of similar design. Similar results were obtained for TNBC.

The analytic tools used here in deriving the signatures, which we have variously named survival scatter plot, hROC, area between curves, or patient selection matrix, alongside the resampling methodology presented, are of general applicability and should be useful in deriving predictive signatures in arbitrary indications, provided corresponding two-arm studies are available.

## Methods

Much of the computational work reported here was performed in R. The package ‘survival’ was used throughout for basic estimation functions such as coxph or Surv. These functions were embedded in custom-built programs written in R and integrated into the Gecko gene expression analysis platform [40]. These programs and all underlying functions are available under project name ‘predSS’ from GitHub (https://github.com/joachimt1/predSS). A detailed description of methods used has been incorporated step by step in the Results section above, as it was felt that this would result in a more organic presentation of the methodology.

## Supplementary information

**Additional file 1.** Data matrix of AFLAME gene expression profiles. Data matrix of AFLAME gene expression profiles in the form of quantile-normalized and batch-corrected log2(read count + 1) values, formatted as a {26,775 × 209} {genes × samples} matrix.

**Additional file 2.** Companion metadata file for AFLAME study. Companion metadata file for AFLAME study with treatment arm, PFS times and censoring status indicated.

**Additional file 3.** LP CRC subtype centroids. Tab-separated-values text file containing the LP CRC subtype centroids.

**Additional file 4.** CMS CRC subtype centroids. Tab-separated-values text file containing the CMS CRC subtype centroids.

**Additional file 5: Supplementary Figure 1.** A stringent threshold selects for a group with fewer patients but with larger treatment benefit. A. Patient selection matrix for the 5-fold cross-validated subC-LP signature with the more stringent decision threshold ∆ξ_c_ = − 1.5 (see Fig. 7 for all definitions). B. Number of patients in each {treatment arm × response group} category. C. hROC showing the split corresponding to ∆ξc = − 1.5. D. KM plot for the *n* = 172 patients in the relatively-resistant group and E., KM plot for the *n* = 37 patients in the sensitive group.

**Additional file 6.** Data matrix of TNBC gene expression profiles. Data matrix of TNBC gene expression profiles in the form of quantile-normalized and batch corrected microarray log2(expression) values, formatted as a {20,756 × 210} {genes × samples} matrix.

**Additional file 7.** Companion metadata file for TNBC study. Companion metadata file for TNBC study with treatment arm, PFS times and censoring status indicated. PFS times are given in units of standard “months” equal to (365.25 / 12) days.

**Additional file 8.** Oxidative stress response genes. List of 101 genes involved in oxidative stress response.

**Additional file 9.** Appendix A. Mathematical definitions for area under hazard ratio curve.

**Additional file 10.** List of Institutional Review Board (IRB) and Ethics Committees for the AFLAME and TNBC studies.

## Data Availability

A table of AFLAME gene expression profiles, containing quantile-normalized and batch-corrected log2(read count + 1) values, formatted as a {26775 × 209} {genes × samples} matrix, alongside a companion metadata file, with patient by patient treatment arm, PFS times and censoring statuses indicated, are available in this article’s Additional files 1 and 2, respectively. A table of TNBC gene expression profiles, containing gene expression profiles in the form of quantile-normalized and batch corrected microarray log2(expression) values, formatted as a {20756 × 210} {genes × samples} matrix, alongside a companion metadata file, with patient by patient treatment arm, PFS times and censoring statuses indicated, are available in this article’s Additional files 6 and 7, respectively. The R-based software for generating the analyses described here is available under project name ‘predSS’ from GitHub (https://github.com/joachimt1/predSS).

## Abbreviations

Abc: Area between curves
CMS: Consensus molecular subtypes
CRC: Colorectal cancer
dLHR: Differential log hazard ratio
FFPE: Formalin-fixed paraffin-embedded
hROC: Hazard ratio receiver operating characteristic
LP: “Laurent-Puig” (classification of CRC subtypes)
PFS: Progression-free survival
PSM: Patient selection matrix
subC: Subtype correlation
TNBC: Triple negative breast cancer
