# Supplementary material for: Construction and optimization of gene expression signatures for prediction of survival in two-arm clinical trials
Source: BMC Bioinformatics. 2020 Jul 25;21:333. doi: 10.1186/s12859-020-03655-7 (PMC7382041; doi:10.1186/s12859-020-03655-7)

Supplementary Figure 1 : **A stringent threshold selects for a group with fewer patients but with larger treatment benefit.** **A**. Patient selection matrix for the 5-fold cross-validated subC-LP signature with the more stringent decision threshold $\Delta\xi_c = -1.5$ (see Figure 7 for all definitions). **B**. Number of patients in each {treatment arm × response group} category. **C**. hROC showing the split corresponding to $\Delta\xi c = -1.5$. **D**. KM plot for the n = 172 patients in the relatively-resistant group and **E**., KM plot for the n = 37 patients in the sensitive group.

Supplementary Figure 1

A threshold $\Delta\xi_c = -1.5$

			prognostic effects					
			sensitivity					
		Tmed	R	S	All(R+S)	n	hR	pR
		control	5.6	4.2	5.6	68	1.525	2.73E-01
predictive	arm	afliberc.	7.5	9.0	7.7	141	0.69	1.14E-01
		n	172	37	209			
		hR	0.564	0.168	0.486			
		pR	1.90E-03	4.12E-04	2.65E-05			
		ALL(0+1)	6.932	8.279	6.932			

B

COUNTS	R	S	All(R+S)
control	59	9	68
afliberc.	113	28	141
ALL(0+1)	172	37	209

C

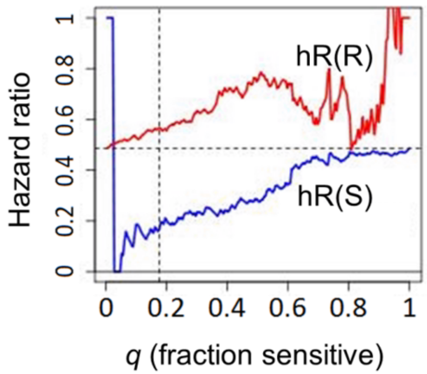

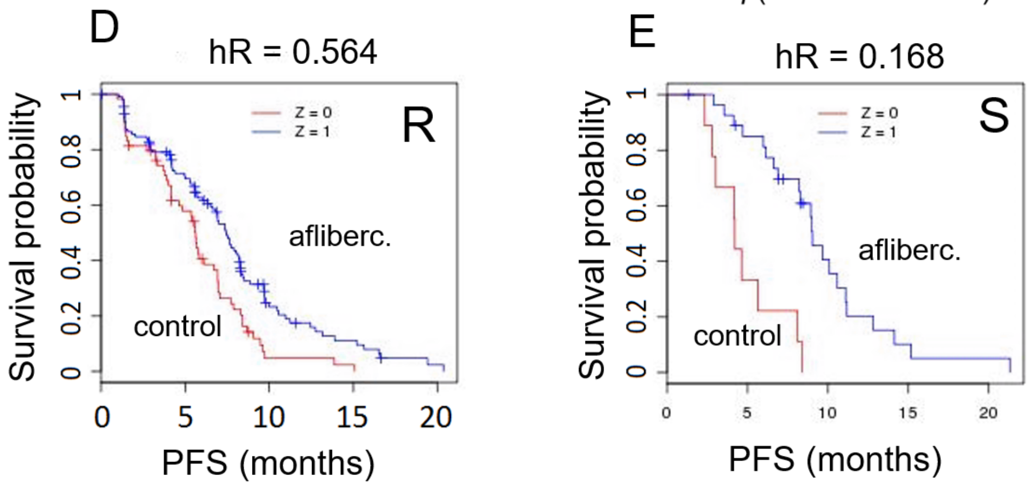

Supplement: Supplementary file 5 — Additional file 5: Supplementary Figure 1. A stringent threshold selects for a group with fewer patients but with larger treatment benefit. A. Patient selection matrix for the 5-fold cross-validated subC-LP signature with the more stringent decision threshold ∆ξc = − 1.5 (see Fig. 7 for all definitions). B. Number of patients in each {treatment arm × response group} category. C. hROC showing the split corresponding to ∆ξc = − 1.5. D. KM plot for the n = 172 patients in the relatively-resistant group and E., KM plot for the n = 37 patients in the sensitive group. [file 12859_2020_3655_MOESM5_ESM.pdf]
