# Supplementary material for: Construction and optimization of gene expression signatures for prediction of survival in two-arm clinical trials
Source: BMC Bioinformatics. 2020 Jul 25;21:333. doi: 10.1186/s12859-020-03655-7 (PMC7382041; doi:10.1186/s12859-020-03655-7)
Supplement: Supplementary file 9 — Additional file 9. Appendix A. Mathematical definitions for area under hazard ratio curve. [file 12859_2020_3655_MOESM9_ESM.pdf]

Appendix A for:

Construction and optimization of gene expression signatures for prediction of survival in two-arm clinical trials

Joachim Theilhaber, Marielle Chiron, Jennifer Dreymann, Donald Bergstrom and Jack Pollard

Appendix A: Area under the hazard ratio curve

Let the parametrized function *h(q)* denote either of the hazard ratios *hR(S)* or *hR(R)* that result when the decision threshold $\Delta\xi_c$ (the parameter) is such that the fraction of patients assigned to the aflibercept-sensitive response group S is q. To compute the area under the curve defined by the function $h = h(q)$, $0 \leq q \leq 1$, we first apply a symmetrizing transformation to *h* defined by the function

$$y(h) = \begin{cases} h-1, & h \leq 1, \\ 1-\dfrac{1}{h}, & h > 1. \end{cases}$$

(A1)

The definition of Eq.(A1) has the properties that y = y(h) and y'(h) are continuous at h = 1 (y(1) = 0, y'(1) = 1), with symmetry y(1/h) = − y(h) (Eq.(A1) also avoids infinities that might occur as h → 0 or h → ∞, if the analytically slightly simpler y(h) = log(h) had been used). After transformation, departures of h above and below h = 1 are equally emphasized, in particular avoiding infinite area when $h \to \infty$. The area A_h under the hazard ratio curve is then defined as

$$A_h = \int_0^1 dq \;\; y(h(q)) \;\; ,$$

(A2)

and substituting Eq.(A2) into Eq.(A1) results in the explicit formula

$$A_h = \int_0^1 dq\ \theta(h\text{-}1)\cdot\left(1\text{-}\frac{1}{h}\right)\ -\ \int_0^1 dq\ \theta(1\text{-}h)\cdot\left(1\text{-}h\right)\ ,$$

(A3)

where $\theta(u)$ is the Heaviside step function ($\theta(u) = 1$ if $u \geq 0$, 0 otherwise).

The statistic defined by Eq.(A3) lies in the range $-1 < A_h < 1$ and thus has a natural scale. In particular, $A_h \rightarrow -1$ if $h(q) \rightarrow 0$ for all values of q in the interval $0 \leq q \leq 1$, and similarly $A_h \rightarrow 1$ if $h(q) \rightarrow \infty$ in the interval.

The usefulness of the symmetrizing transformation of Eq.(A1) is perhaps made clearer if we consider interchanging the roles of the treatment arms, with control arm now considered the "aflibercept" arm, and the aflibercept arm now considered the "control" arm. Let the parametrized hazard ratio of risk under "aflibercept" to "control" treatments be denoted by $\tilde{h}(\tilde{q})$, where $\tilde{q}$ is the fraction of patients sensitive to "aflibercept" treatment. By definition of the treatment arms, we have $\tilde{q} = 1 - q$, and

$$\tilde{h}(q) = \frac{1}{h(\tilde{q})} = \frac{1}{h(1-q)}\ .$$

(A4)

Introducing Eq.(A4) into Eq.(A2), using the relation $y(1/h) = -y(h)$, and changing integration variable q to $q' = 1 - q$, we obtain the symmetry

$$A_{\tilde{h}} = -A_h\ ,$$

(A5)

correctly reflecting the reversal of the roles of the treatment arms.

To generate a general figure of merit for a given model-based patient stratification, we compute the area between curves A_{bc} , defined by

$$A_{bc} = A_{h_R} - A_{h_S}\ ,$$

(A6)

where hR = hR (q) refers to the hazard ratio that obtains as a function of q for the relatively-resistant population (right-hand side of boundary in **Figure 3)** and hS = hS(q) refers to the hazard ratio that obtains as a function of q for the sensitive population (left-hand side of boundary in **Figure 3**).

Note that an ideal patient stratification scheme would be one that minimizes hS(q) as much as possible for all values of q (so that $A_{hS} \rightarrow -1$ as much as possible), and simultaneously maximizes hR (q) as much as possible for all values of q (so that $A_{hR} \rightarrow 1$ as much as possible), insuring that each patient group gets assigned the most appropriate treatment arm. Thus "large" values of A_{bc} are indicative of good performance of the predictive model.

In practice, A_{h_R} and A_{hS} are never independent, so that the strict numerical upper bound of $|A_{bc}| \leq |A_{h_R}| + |A_{h_S}| \leq \max|A_{h_R}| + \max|A_{h_S}| = 2$, is never reached. As an idealized example, consider a population such that for one half of the patients, aflibercept to control arm hazard ratio is given by $h_1 < 1$, and for the other half the corresponding hazard ratio is $1 / h_1$. We assume the existence of a continuous univariate biomarker with some finite range, which perfectly separates the two populations. For this ideal case, it can be shown that the area between curves is given approximately by

$$A_{bc} = \frac{3}{2}\left(1 - h_1\right)$$

(A7)

In particular, for extreme differences in survival according to treatment arm within each group, $h_1 \ll 1$, we have $A_{bc} \approx 1.5$, which can be considered an upper bound to values of Abc .
