# Supplementary material for: Construction and optimization of gene expression signatures for prediction of survival in two-arm clinical trials
Source: BMC Bioinformatics. 2020 Jul 25;21:333. doi: 10.1186/s12859-020-03655-7 (PMC7382041; doi:10.1186/s12859-020-03655-7)
Supplement: Supplementary file 10 — Additional file 10. List of Institutional Review Board (IRB) and Ethics Committees for the AFLAME and TNBC studies. [file 12859_2020_3655_MOESM10_ESM.docx]

List of Institutional Review Board (IRB) and Ethics Committees for studies analyzed in the article “Construction and optimization of gene expression signatures for prediction of survival in two-arm clinical trials” by J. Theilhaber et al.

**AFLAME clinical study (aflibercept) [NCT01661270]**

1. Ethics Committee, Beijing cancer hospital, No. 52, Fucheng Road, Haidian district, Beijing, CHINA
2. Ethics Review Committee of Cancer hospital Chinese academy of medical science No.17 Panjiayuan south Chaoyang district, Beijing CHINA.
3. Ethics Review Committee of the 307th Hospital of Chinese People‘s Liberation Army/No.8, East street, Fengtai District, Beijing City, CHINA
4. Chinese PLA General Hospital, No.28, Fuxing Road ,Haidian District, Beijing, CHINA
5. Ethics Review Committee of Jiangsu Province Hospital/ No300,Guangzhou Road,Nanjing, CHINA
6. The first Hospital of China Medical University Medical Ethics committee ,No.155 Nanjing North Street Heping District,Shenyang,110001,CHINA
7. Harbin Medical University Cancer Hospital Medical Ethics committee,No.150 Haping Road NangangDistrict,Harbin,150081, CHINA
8. Ethic Committee of West China Hospital of Sichuan University/ No. 37, Guoxuexiang, Chengdu, 610041, CHINA
9. Ethic Committee of First Affiliated Hospital of 4th Military Medical University / No.127, Changle Road (West), Xi’an, 710032, CHINA
10. Ethics Committee of Clnical Trail, Huazhong University of Science and Technology No.13,Hangkong Road, Wuhan, CHINA
11. Ethics Committee of Clnical Trail, Huazhong University of Science and Technology No.13,Hangkong Road, Wuhan, CHINA
12. Ethic Committee of First Affiliated Hospital of 3rd Military Medical University, PLA No, 30, Gaotanyanzheng Road, Chaping District, Chongqing, CHINA
13. Ethics Review Committee of Fujian provincial cancer hospital No.91 Fumalu, Jin’an district, Fuzhou, CHINA
14. No.135, Nan-Hsiao St., Changhua 500, TAIWAN
15. No.201, Sec. 2, Shih-Pai Road, Taipei 112, TAIWA
16. No.7, Chung Shan South Rd., Taipei 100, TAIWAN
17. HKU/HA HKW IRB Room 901, Administration Block, Queen Mary Hospital, HONGKONG
18. Kobe City Medical Center General Hospital IRB 2-1-1, Minatojimaminamimachi, Chuoku, Kobe-Shi 650-0047 Hyogo JAPAN
19. Saitama Cancer Center IRB 780 Ina-machi Komuro, KitaadachiGun 362-0806 Saitama JAPAN
20. Kansai Rosai Hospital IRB 3-1-69 Inabaso, Amagasaki-Shi 660-8511 Hyogo JAPAN
21. Kumamoto University Hospital IRB 1-1-1 Honjo, Chuo-ku, Kumamoto-Shi 860-8556 Kumamoto JAPAN
22. Aichi Medical University Hospital IRB 1-1 Yazakokarimata, Nagakute-Shi 480-1195 Aichi JAPAN
23. Gifu University Hospital IRB 1-1 Yanagido, Gifu-Shi 501-1194 Gifu JAPAN
24. Tokyo Metropolitan Cancer and Infectious Diseases Center Komagome Hospital IRB 3-18-22, Honkomagome Bunkyo-Ku 113-8677 Tokyo JAPAN
25. Medical Hospital, Tokyo Medical And Dental University IRB 1-5-45 Yshima, Bunkyo-Ku 113-8519 Tokyo JAPAN
26. Kochi Health Sciences Center IRB 2125-1 Ike, Kochi-Shi 781-8555 Kochi JAPA
27. Osaka Medical College Hospital IRB 2-7, Daigakumachi, Takatsuki-Shi 569-0801 Osaka JAPAN
28. Singhealth Centralised Institutional Review Board (CIRB) B 168 Jalan Bukit Merah #06-08 Tower 3 Connection One SINGAPORE 150168
29. National Healthcare Group Domain Specific Review Board (DSRB) B 3 Fusionopolis Link #03-08 Nexus@one-north SINGAPORE 138543

**TNBC clinical studies (iniparib) [NCT00813956, NCT01045304]**

1. Supervisory Ethics Committee, Stanford Comprehensive Cancer Center, Stanford, California, United States, 94305
2. Ethics Committe, PrECOG, Philadelphia, Pennsylvania, United States, 19103
3. IRB, Department of Medical Oncology, Institut Curie, Paris, France
4. IRB, Centre Eugène Marquis, Avenue de la bataille Flandres-Dunkerque, CS 44229, 35042, Rennes Cedex, France
5. CIRB Institut Bergonié, Univ. Bordeaux, INSERM U1218, INSERM CIC1401, Bordeaux, France
6. Ethics Committee, Department of Clinical Oncology, Hospital Clínico Universitario Virgen de la Victoria, Málaga, Spain
7. Ethics Committee/Oncology Medicine Department, Institut Jules Bordet, Université Libre de Bruxelles, Brussels, Belgium
8. IRB, Medical Oncology Department, Centre Georges François Leclerc, Dijon, France
9. Ethics Committee, Department of Medical Oncology, Centre Paul Strauss, Strasbourg, France
10. IRB-II/Medical Oncology Dept Tenon Hospital, Inserm U938, Institut Universitaire de Cancérologie APHP-Sorbonne Université, Paris, France
11. Ethics Committee, Department of Medical Oncology, Erasmus MC Cancer Institute, Rotterdam, The Netherlands
12. CIRB, Department of Medical Oncology, IRCCS OSR, San Raffaele, Milan, Italy
13. CIRB, Department of Medical Oncology, Peter MacCallum Cancer Centre and Royal Melbourne Hospital, The Walter and Eliza Hall Institute of Medical Research, Melbourne, VIC, Australia
14. IRB, Department of Medical Oncology, University Hospital Gasthuisberg, Catholic University of Leuven, Leuven, Belgium
